# Phosphatidylinositol-3-phosphate mediates Arc capsid secretion through the multivesicular body pathway

**DOI:** 10.1073/pnas.2322422121

**Published:** 2024-08-23

**Authors:** Kritika Mehta, Henry Yentsch, Jungbin Lee, Yeeun Yook, Kwan Young Lee, Tianyu Terry Gao, Nien-Pei Tsai, Kai Zhang

**Affiliations:** ^a^Department of Biochemistry, University of Illinois Urbana-Champaign, Urbana, IL 61801; ^b^NSF Science and Technology Center for Quantitative Cell Biology (STC-QCB) Center, University of Illinois Urbana-Champaign, Urbana, IL 61801; ^c^Department of Molecular and Integrative Physiology, University of Illinois Urbana-Champaign, Urbana, IL 61801; ^d^Center for Biophysics and Quantitative Biology, College of Liberal Arts and Sciences, University of Illinois Urbana-Champaign, Urbana, IL 61801; ^e^Neuroscience Program, College of Liberal Arts and Sciences, University of Illinois Urbana-Champaign, Urbana, IL 61801; ^f^Beckman Institute for Advanced Science and Technology, University of Illinois Urbana-Champaign, Urbana, IL 61801; ^g^Cancer Center at Illinois, University of Illinois Urbana-Champaign, Urbana, IL 61801

**Keywords:** activity-regulated cytoskeleton-associated protein, intercellular RNA transfer, phospholipids, multivesicular body, virus-like capsid

## Abstract

Activity-regulated cytoskeleton-associated protein (Arc/Arg3.1) is an immediate early gene that plays a vital role in learning and memory. Arc protein has structural and functional properties similar to viral Group-specific antigen (Gag) protein and mediates the intercellular RNA transfer through virus-like capsids. However, the regulators and secretion pathway through which Arc capsids maneuver cargos are unclear. Here, we identified that phosphatidylinositol-3-phosphate (PI3P) mediates Arc capsid assembly and secretion through the endosomal–multivesicular body (MVB) pathway. Indeed, reconstituted Arc protein preferably binds to PI3P. In HEK293T cells, Arc forms puncta that colocalize with FYVE, an endosomal PI3P marker, as well as Rab5 and CD63, early endosomal and MVB markers, respectively. Superresolution imaging resolves Arc accumulates within the intraluminal vesicles of MVB. CRISPR double knockout of RalA and RalB, crucial GTPases for MVB biogenesis and exocytosis, severely reduces the Arc-mediated RNA transfer efficiency. RalA/B double knockdown in cultured rat cortical neurons increases the percentage of mature dendritic spines. Intake of extracellular vesicles purified from Arc-expressing wild-type, but not RalA/B double knockdown, cells in mouse cortical neurons reduces their surface GlutA1 levels. These results suggest that unlike the HIV Gag, whose membrane targeting requires interaction with plasma-membrane-specific phosphatidyl inositol (4,5) bisphosphate (PI(4,5)P2), the assembly of Arc capsids is mediated by PI3P at endocytic membranes. Understanding Arc’s secretion pathway helps gain insights into its role in intercellular cargo transfer and highlights the commonality and distinction of trafficking mechanisms between structurally resembled capsid proteins.

## Significance

Activity-regulated cytoskeleton-associated protein (Arc) serves as a pivotal regulator for neuronal communication, influencing both local synaptic transmission and long-range intercellular signaling through direct material exchange. However, the mechanism governing the assembly and secretion pathways of Arc’s virus-like capsids remains elusive. Our study integrates biochemical analyses, cell biology methodologies, and cutting-edge superresolution imaging techniques to unveil that Arc relies on the endosomal–multivesicular body axis for capsid assembly and secretion mediated by endosomal PI3P. Understanding the cellular machinery orchestrating Arc capsid assembly and secretion sheds light on how structurally similar capsid proteins evolve distinct trafficking strategies.

On average, each neuron cell forms approximately 1,000 synapses, proximal connections between cells that facilitate information relay across cells mediated by neurotransmitters. Neuronal activity at a synapse could stimulate a long-distance synapse-to-nucleus communication to activate the transcription of immediate early genes in the stimulated cells. One such actively transcribed gene is activity regulated cytoskeleton-associated protein (Arc/Arg3.1), whose mRNA is transported to neuronal dendrites in response to synaptic activity (1–3). The local translation of Arc mRNA produces Arc protein, which mediates synaptic protein trafficking and modifies local proteome, leading to morphological and functional changes of synaptic strength between associated cells. Such a “fire-together, wire-together” Hebbian synaptic plasticity is believed to underpin molecular mechanisms underlying learning, memory formation, and long-term memory consolidation.

While synapse-to-nucleus communication represents a unique mode of action for long-distance communication in polarized neuronal cells, other mechanisms exist in nature to facilitate proximity-independent communication between mammalian cells (4–19). For example, retroviruses package their genome into capsids, a supramolecular structure that could undergo exocytosis from a parent cell, diffuse through the extracellular space, and infect a recipient cell. Such long-distance delivery of capsids between cells necessitates infrastructure for stability and sufficient volume. Extracellular vesicles (EVs) are among such infrastructures that play an effective role in intercellular communications (20–26). The spatial and temporal release of EVs into the extracellular space is regulated by the cellular machinery involved in its biogenesis and exocytosis (22). Exosomes are a class of EVs that employ the multivesicular body (MVB) pathway to generate membrane-bound EVs inside the cell’s cytoplasm (27). Microvesicles, on the other hand, release via outward budding of the plasma membrane (28).

It was not expected that such a capsid-mediated pathway could have implications in neuronal communication until recently when Arc was found to regulate intercellular RNA transfer. In this process, Arc proteins assemble into high-order, virus-like capsids encapsulating its RNA, exocytose from the parent cell, and infect a remote recipient neuron (14, 15). Arc’s role in the mediation of intercellular RNA transfer is not completely surprising, considering Arc is a retrotransposon-derived gene, but structurally resembled protein could evolve distinct trafficking pathways with unique regulators or mediators (29–31). Phospholipids play a crucial role in retrovirus capsid assembly and trafficking. For example, stable attachment of the HIV Gag to the membrane requires its specific interaction with phosphatidylinositol-4,5-bisphosphate (PI(4,5)P2) enriched at the plasma membrane (32, 33). However, it is unclear whether phospholipids regulate Arc capsid biogenesis and trafficking (34).

Here, we employ a combination of genetic, imaging, and biochemical analysis to understand the biogenesis and trafficking of Arc-containing capsids. Unlike HIV particles, which assemble proximal to the plasma membrane, Arc capsid assembly, and secretion proceed through the endosomal–MVB pathway. Understanding Arc capsid assembly and trafficking will shed light on the commonality and distinction of molecular machinery regulating structurally resembled capsid proteins. Knowledge of Arc intercellular trafficking could provide a better understanding of brain physiology and pathology, as it has been found that various Arc mutants occur in patients with neurological and neuropsychiatric diseases such as Alzheimer’s disease, autism spectrum disorder, and Schizophrenia (35, 36).

## Results

### HaloArc Mediates Intercellular RNA Transfer and Protein Expression.

**Fig. 1.**
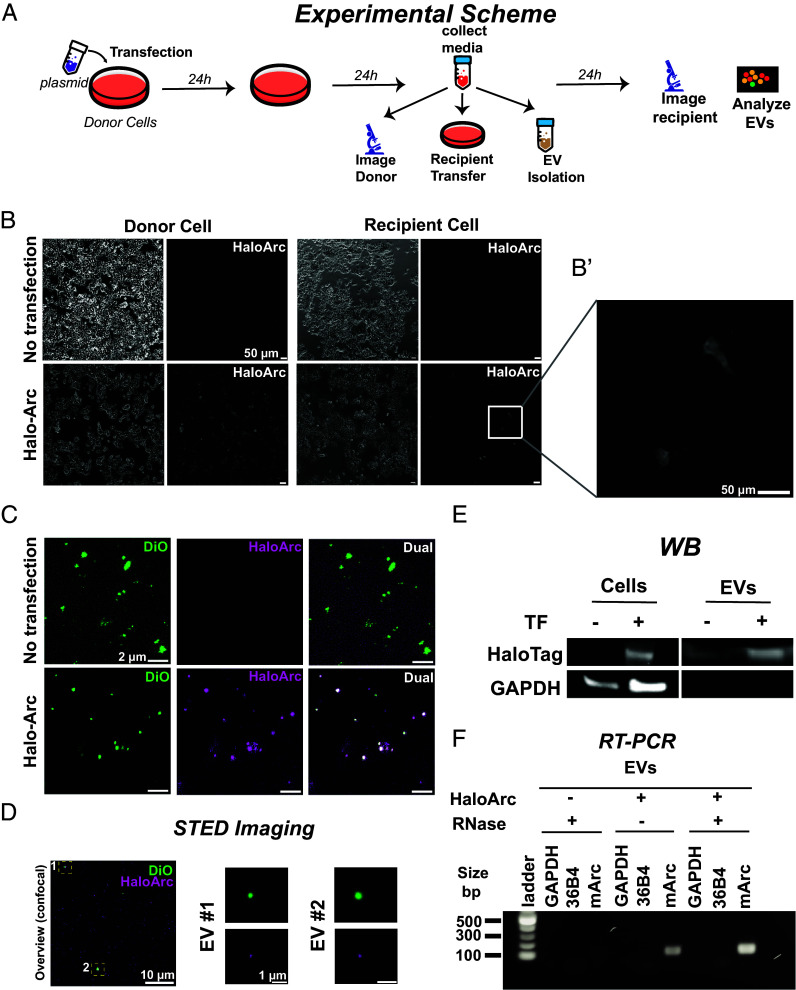
Halo-Arc can transfer between HEK293T cells. (*A*) Workflow of Arc-mediated intercellular RNA transfer. (*B*) Donor-recipient assay using nontransfected (*Top*) or HaloArc-expressing (*Bottom*) cells. (*B*′) HaloArc expression in recipient cells. (Scale bar, 50 µm.) (*C*) Epifluorescence imaging of EVs isolated from the conditioned medium of untransfected (*Top*) or HaloArc-transfected (*Bottom*) cells labeled with 5 μM DiO (green) and 100 nM JF549 Halo ligand (magenta). (Scale bar, 2 µm.) (*D*) Representative 2D-STED image of an EV stained with DiO (green, confocal mode) and JF-646 halo ligand (magenta, STED mode) capturing HaloArc resides inside the EVs. (Scale bar, 500 nm.) (*E*) Western blot analysis of HaloArc expression in cell lysates (*Left*) or EVs (*Right*). (*F*) Reverse transcription PCR analysis of HaloArc mRNA inside the EV fraction with and without RNase treatment.

To visualize the Arc protein dynamics in live cells, we constructed HaloArc, a plasmid containing mouse Arc with an N-terminal fusion of HaloTag, flanked by the 5′-UTR and 3′-UTR of the Arc mRNA (*SI Appendix*, Fig. S1*A*). UTRs were included because of their potential function in mediating intercellular RNA transfer (14). We first confirmed that this synthetic Arc maintains the capacity to transfer RNA between HEK293T cells, which express no endogenous Arc but allow for validation of Arc RNA transfer shown in previous work (14). We developed the donor-recipient assay as depicted in Fig. 1*A*, in which the conditioned medium from HaloArc-expressing donor cells was incubated with nontransfected recipient cells to confirm intercellular RNA transfer and expression. In parallel, EV were extracted from part of the conditioned medium for further analysis. A successful HaloArc-mediated donor-recipient RNA transfer assay fulfills the following criteria: 1) up-regulated HaloArc protein expression in the recipient cells, 2) enrichment of HaloArc protein in the EVs, and 3) enrichment of HaloArc RNA in the EVs.

Expression of HaloArc in the donor cells was confirmed by western blot with a primary antibody against HaloTag (75 kD) (*SI Appendix*, Fig. S1*B*). Expression of HaloArc was also confirmed by fluorescence imaging, where JF549-labeled HaloTag ligand was used to label HaloTag in live cells. In donor HEK293T cells transfected with HaloArc, more than 80% of cells showed strong fluorescence from JF549. Nontransfected cells showed no detectable fluorescence (Fig. 1 *B*, *Left*). When the same labeling procedure was applied to recipient cells incubated with the conditioned medium of transfected donor cells, a similar enhancement of fluorescence was found, indicating successful expression of HaloArc in the recipient cells (criterion #1) (Fig. 1 *B*, *Right* and Fig. 1*B ′*). We further confirmed that Arc-containing EVs were membrane-bound by costaining Arc with DiO, a lipophilic tracer with green fluorescence. Arc-positive EVs unanimously showed green fluorescence, indicating the presence of a membrane structure (Fig. 1*C*). The average fluorescence intensity of Arc-expressing recipient cells was slightly less than that of Arc-expressing donor cells. However, both were significantly above the basal level from the non-Arc-expressing cells in the same culture (*SI Appendix*, Fig. S1*C*). To determine the relative location of the Arc protein with respect to the EV membrane, we applied stimulated emission depletion (STED) superresolution imaging to resolve Arc proteins. HaloArc proteins (JF549, STED mode) localized within the membrane structure (DiO, confocal mode), consistent with a membrane-bound exosome encapsulating Arc capsid (Fig. 1*D*). The average full-width half-maximum of the Arc capsids’ diameter is 153 ± 42 nm (n = 5, *SI Appendix*, Fig. S1*D*). Western blot analysis of purified EVs reveals the presence of HaloArc, meeting criterion #2 (Fig. 1*E*). Finally, RT-PCR measurement reveals that mArc RNA resides in EVs, likely within the capsids, because RNase treatment did not degrade the RNA, fulfilling criterion #3 (Fig. 1*F*). We noted that the band intensity of the RNase(+) sample is higher than that of the RNase(−) sample, likely because RNase treatment enriched HaloArc RNA by degrading other EV-residing RNAs outside the capsids. These results confirmed that HaloArc functions similarly to endogenous Arc in mediating the intercellular transfer of RNA, which can be expressed in the recipient cells.

### HaloArc Confines into Slow-diffusing, Membrane-Associated Puncta in Mammalian Cells.

**Fig. 2.**
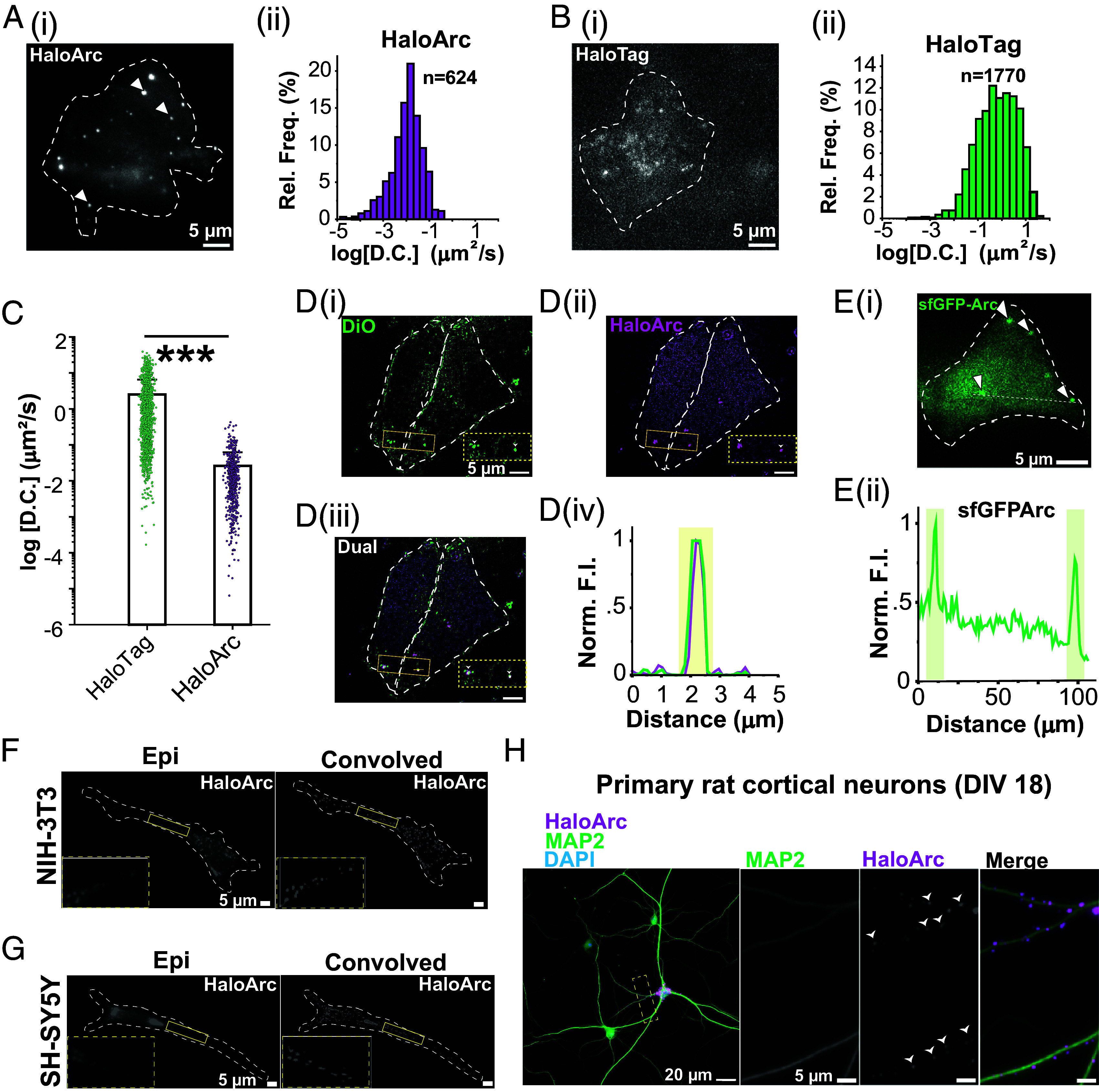
Halo-Arc assembles into membrane-associated clusters in live cells. (*A*) (i) A representative HILO image of a HEK293T cell expressing HaloArc stained in JF549 halo ligand (arrows indicate the HaloArc clusters). (ii) Histogram for the diffusion coefficient for tracked halo-Arc particles (n = 624 particles; N = 3 experiments). (*B*) Same as *A* but for HaloTag only (n = 1,770 particles; N = 3 experiments). (*C*) Scatter plot of the diffusion coefficients of HaloArc and HaloTag particles (Mann–Whitney test, ***P < 0.001). (*D*) (i and ii) Representative convolved epifluorescence image of transfected HEK293T cells stained with DiO (*Left*, green) and JF549 HaloTag ligand for HaloArc (*Right*, magenta). (iii) Merged image of (i and ii). Arrowheads indicate HaloArc clusters localized within DiO-stained vesicles. (iv) Intensity profile across the ROI in (iii). (Scale bar, 5 µm.) (*E*) (i) Representative epifluorescence image of a HEK293T cell expressing sfGFP-Arc (arrows indicate Arc clusters). (ii) Intensity profile across the ROI in (i). (*F*) Representative epifluorescence (*Left*) and convolved (*Right*) images of a stained NIH3T3 fibroblast expressing HaloArc. *Inset*: HaloArc cluster in cells. (*G*) Same as *F* but in SH-SY5Y cells. (*H*) Immunofluorescence staining of MAP2 (neuronal marker) overlaid with HaloArc (magenta) and DAPI (nucleus).

We reasoned that snapshots of the subcellular distribution and morphology of HaloArc could imply their protein trafficking pathways. A home-built highly inclined and laminated optical sheet (HILO) microscopy was used for single-molecule imaging inside cells (37). When expressed and fluorescently labeled in HEK293T cells, HaloArc formed slow-diffusing, almost static puncta in the cytoplasm (Fig. 2*A* and Movie S1). This structure was not an artifact of HaloTag because fluorescently labeled HaloTag alone showed typical fast diffusion in HEK293T cells (Fig. 2*B* and Movie S2). We collected diffusion trajectories from 1,770 HaloTag molecules and 624 HaloArc puncta and constructed a histogram of their diffusion coefficients (Fig. 2 *A*(ii) and *B*(ii)). The probability distribution of both histograms appeared to be lognormal with similar widths. The mean diffusion coefficient of HaloTag alone is 1.0 μm^2^/s, almost two orders of magnitude larger than that of HaloArc (0.01 μm^2^/s) (Fig. 2*C*). The HaloArc puncta are significantly brighter than HaloTag alone, indicating the clustering of Arc molecules. HaloArc puncta are associated with the membrane because DiO-positive puncta colocalized with HaloArc (Fig. 2*D*). When HaloTag was replaced by superfolder GFP (sfGFP), sfGFP-Arc also formed large puncta in HEK293T cells, indicating that puncta formation is not likely caused by fusion proteins (Fig. 2*E*). Compared to HaloArc, sfGFP-Arc shows a more apparent cytosolic diffusive population and lower contrast of Arc puncta, indicating both diffusive and puncta-enriched subpopulations. The high contrast of HaloArc is likely because fluorescence underlabeling of HaloArc highlights the puncta-enriched population. We further confirmed that HaloArc forms puncta in various mammalian cell lines, such as the NIH3T3 fibroblasts (Fig. 2*F*), SH-SY5Y neuroblastoma (Fig. 2*G*), and the primary rat cortical neuronal cultures transduced by lentivirus encoding HaloArc (Fig. 2*H* and *SI Appendix*, Fig. S1*E*).

### Recombinant Arc Proteins Assemble into Capsids.

To further understand Arc assembly, we purified Arc from bacteria using GST affinity chromatography (*SI Appendix*, Fig. S2 *A* and *B*). Further analysis by size exclusion chromatography (SEC) showed that Arc eluted into three peaks with the estimated sizes of 670, 200, and 100 kDa (*SI Appendix*, Fig. S2*C*). All three fractions were pooled together and used for protein characterization (*SI Appendix*, Fig. S2*D*). Mass spectroscopy further confirms the identity of purified Arc (*SI Appendix*, Fig. S2 *E* and *F*). Western blot analysis with purified Arc protein showed a single band at approximately 50 kDa probed by an Arc primary antibody (*SI Appendix*, Fig. S2*G*). Consistent with previous work (14), incubation with 500 mM sodium phosphate buffer shifted the size of Arc from 14 nm to 30 nm in diameter as measured by dynamic light scattering (DLS) (*SI Appendix*, Fig. S3*A*). Arc protein treated with phosphate and mixed with RNA in a 10:1 (w/w) ratio was also imaged using negative staining transmission electron microscopy (TEM), which clearly revealed that Arc formed capsid structure with an average diameter of 32.9 nm (n = 201 particles), consistent with DLS measurement (*SI Appendix*, Fig. S3 *B*–*D*). Capsid induction appeared to be independent of RNA sequences, as RNAs from whole cells, GFP, and Arc all induce capsid formation in the high phosphate buffer (*SI Appendix*, Fig. S3*E*). These results confirmed that purified Arc protein can assemble into capsid-like structures.

### Arc Proteins Are Associated Preferably with Phosphatidylinositol-3-phosphate.

**Fig. 3.**
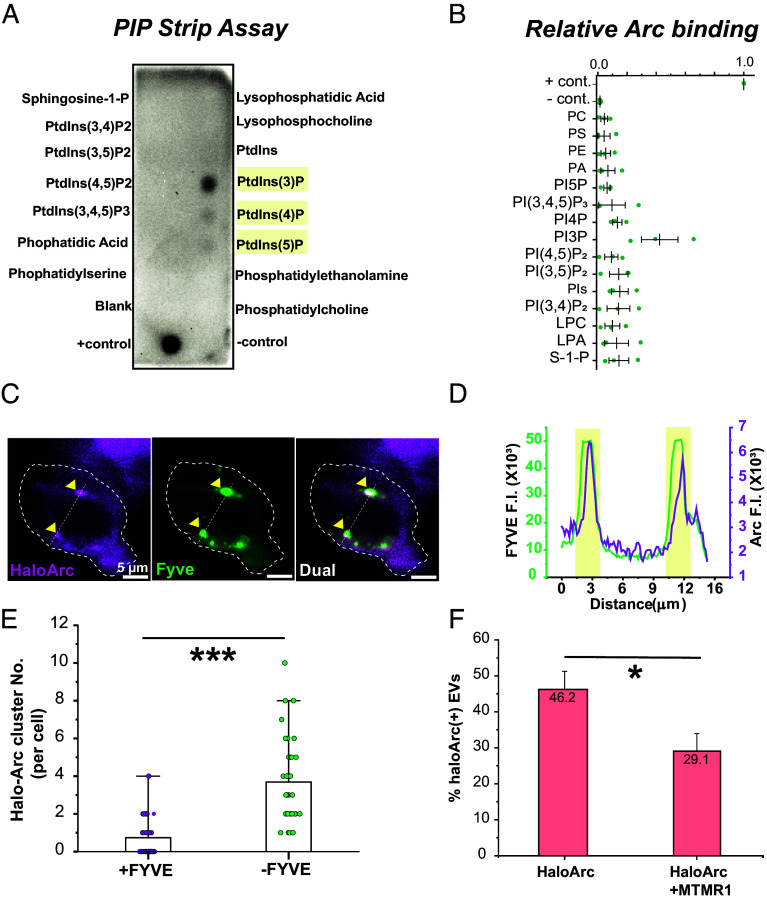
Arc protein interacts with PI3P. (*A*) Results of PIP-strip lipid-overlay assay showing preferable Arc binding to monophosphorylated phosphoinositides (highlighted in yellow). Arc protein is probed using the Arc antibody. (*B*) Quantification of PIP-strip signal intensity in *A*. (*C*) Representative 2-color HILO image of a HEK293T cell coexpressing EGFP-2×FYVE (green; a PI3P marker) and HaloArc (magenta). Arrowheads mark HaloArc clusters colocalized with the Fyve domain (dual). (Scale bar, 5 μm.) (*D*) Profile of fluorescence intensity along the line in *C*. Colocalized clusters are shown in yellow. (*E*) Quantification of the count of HaloArc clusters per cell under the HILO field with (pink; n = 34 cells) or without (green; n = 35 cells) FYVE cotransfection. (*F*) Percentages of HaloArc-positive EVs produced by cells expressing HaloArc or HaloArc+MTMR1, normalized by the number of all EVs stained by DiO. (Mann–Whitney test; *P < 0.05 and **P < 0.001).

Phospholipids play a crucial role in regulating protein trafficking in live cells. Distinct pools of phospholipids reside in the membranes of different intracellular vesicles. We reason that Arc assembly inside cells could be mediated through phospholipids. To determine whether Arc selectively binds to phospholipids, we performed a lipid–protein interaction assay by incubating purified Arc protein with a membrane lipid strip, a membrane prespotted with 15 different types of lipids. Intriguingly, Arc proteins are preferably associated with monophosphorylated phosphatidylinositol, phosphatidylinositol-3-phosphate (PI3P), phosphatidylinositol-4-phosphate (PI4P), and phosphatidylinositol-3-phosphate (PI5P) (Fig. 3*A*). In particular, PI3P is associated with Arc with approximately twofold and threefold stronger affinity than PI4P and PI5P, respectively (Fig. 3*B*).

Motivated by this result, we tested whether Arc binds to PI3P in cells. The FYVE finger (zinc finger originally observed in Fab1p, YOTB, Vac1p, and EEA1) protein functions in the membrane recruitment of cytosolic proteins by binding to PI3P, which is found primarily on endosomes and is commonly used as a PI3P marker in cells (38). We cotransfected HaloArc with EGFP-2×FYVE in HEK293T cells and performed two-color HILO imaging (*SI Appendix*, Fig. S4*A* and Movies S3 and S4). Consistent with the PIP-strip result, we observed colocalization between HaloArc and EGFP-2×FYVE (Fig. 3 *C* and *D*), indicating Arc’s preferable binding to PI3P. Curiously, the number of Arc puncta (no FYVE, Arc clusters = 3.7 ± 2.3; n = 34 cells) is reduced when cotransfected with EGFP-2×FYVE (transfected with FYVE, Arc clusters = 0.7 ± 1.0; n = 35 cells), which implies that FYVE’s competitive binding to PI3P affects Arc puncta formation (Fig. 3*E*). Interestingly, a previous study on PIP-regulated gephyrin clustering also presented a similar reduction in gephyrin clusters with prolonged FYVE overexpression, possibly due to competitive binding of FYVE to membrane PI3P (39). To further understand the effect of PI3P on Arc exocytosis, we coexpressed MTMR1, a PI3P phosphatase, along with HaloArc and found that coexpression of MTMR1 significantly reduced HaloArc secretion (Fig. 3*F*). These results suggest that PI3P may mediate the endosomal entry of Arc protein inside cells.

To determine whether PI3K activity is necessary for Arc association with the endosomal membrane, we inhibited PI3K activity with Wortmannin. A dose-dependent inhibition of PI3K, indicated by failing to phosphorylate serine 473 of endogenous AKT, confirmed that 5 μM of Wortmannin is sufficient to block PI3K activity in HEK293T cells (*SI Appendix*, Fig. S5*A*). Thus, we chose this concentration for follow-up experiments. Compared to DMSO treatment, when treated with Wortmannin, HEK293T cells expressing HaloArc showed a significant reduction of HaloArc clusters (*SI Appendix*, Fig. S5 *B*–*D*), indicating PI3K activity is necessary for mediating Arc association with the endosomal membrane.

### HaloArc Clusters Colocalize with Markers for Early Endosome and MVB in Mammalian Cells.

**Fig. 4.**
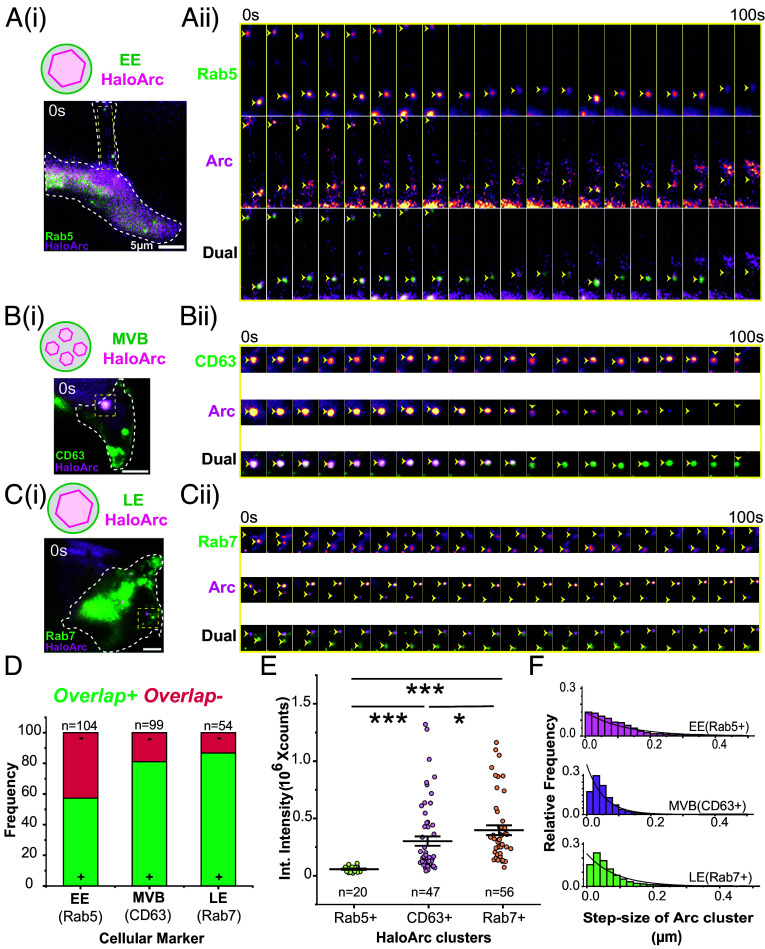
Arc clusters exist inside different endosomal species. (*A*) (i) A representative 2-color HILO image of HEK293T cells cotransfected with HaloArc (magenta) and EGFP-Rab5 (green). (ii) Time-lapse images of the selected ROI in (i). Arrowheads indicate HaloArc colocalized with Rab5+ vesicles. (*B*) (i) A representative 2-color HILO image of HEK293T cells cotransfected with HaloArc (magenta) and sfGFP-CD63 (green). (ii) Time-lapse images of the selected ROI in (i). Arrowheads indicate HaloArc colocalized with CD63+ vesicles. (*C*) (i) A representative 2-color HILO image of a HEK293T cell cotransfected with HaloArc (magenta) and EGFP-Rab7 (green). (ii) Time-lapse images of the selected ROI in (i). Arrowheads indicate HaloArc colocalized with Rab7+ vesicles. (*D*) Quantification for the frequency of overlap between HaloArc clusters and each vesicle marker (Rab5: early endosome, n = 104 clusters; CD63: multivesicular body, n = 99 clusters; and Rab7: late endosome, n = 54 clusters). (*E*) Average integrated fluorescence intensities of HaloArc clusters overlapped with each vesicle marker (Rab5, n = 20; CD63, n = 56; Rab7, n = 47). (*F*) Step size histogram of HaloArc cluster overlapped with each vesicle marker. (Mann–Whitney test; *P < 0.05 and ***P < 0.001). (Scale bar, 5 μm for all images.)

PI3P is enriched on the limiting membrane of early endosomes and intraluminal vesicles within MVBs (38); therefore, we proceeded to determine the identity of Arc-associated vesicles along the endosomal pathways. We carried out colocalization experiments between HaloArc and various markers of the endo-lysosomal system, including EGFP-Rab5 (early endosomal), EGFP-Rab7 (late endosomal and MVB), and sfGFP-CD63 (MVB). We used dual-color single-particle tracking to follow the trafficking trajectory of vesicles under both emission channels. When the puncta in both green (vesicle marker) and red (Arc) channels move together during the whole 200-s data acquisition time, markers for both channels are more likely to reside on the same puncta (Fig. 4 *A*–*C* and Movies S5–S7).

We then calculated the frequency of overlap by dividing the number of green-red double-positive particles by that of all red-positive particles (Arc) collected from all imaged cells. This metric indicates the distribution of the puncta-enriched Arc subpopulation along the endocytic-MVB pathway. We found that the frequency of overlap is 58.5% for Rab5^+^-Arc^+^ double-positive vesicles (n = 104 vesicles; N = 17 cells), 81.0% for CD63^+^-Arc^+^ vesicles (n = 99 vesicles; N = 67 cells), and 86.7% for Rab7^+^-Arc^+^ vesicles (n = 54 vesicles; N = 25 cells) (Fig. 4*D*). We noticed these percentages do not add to 100%, indicating these markers are not mutually exclusive. Indeed, previous work showed that during endosomal maturation, incomplete Rab5 detachment and Rab7 recruitment could result in Rab5^+^-Rab7^+^ double-positive endosomes (40). However, these high overlap frequencies suggest that the puncta-enriched Arc uses the endocytic-MVB pathway for intracellular trafficking. To further support this idea, we examined sfGFP-Arc alongside another MVB marker protein, mCherry-TSG101, and found a frequency of overlap of 94.7% (n = 20 vesicles, *SI Appendix*, Fig. S6 *A*, *B*, and *E* and Movie S8). Furthermore, colocalization studies with EGFP-Rab7 and mCherry-TSG101 revealed a 93.3% colocalization of both markers (n = 30 vesicles, Fig. S6 *C*–*E* and Movie S9), suggesting that both Rab7 and TSG101 are effective markers for MVBs.

Intensity analysis shows that early endosome-associated Arc puncta (colocalized with Rab5, n = 20) showed a lower fluorescence intensity than those associated with late endosomes or MVBs (Rab7, n = 47; CD63, n = 56) (Fig. 4*E* and *SI Appendix*, Fig. S6*F*). Analysis of HaloArc puncta step size (100 ms frame rate) shows that early endosome-associated HaloArc has a larger step size than those colocalized with MVBs and late endosomes (Fig. 4*F*). These results indicate that Arc proteins might acquire higher-order oligomeric states as they proceed through the endosome–MVB pathway.

It is known that Vps4 ATPase and the ESCRT (endosomal-sorting complex required for transport) protein complexes work synergistically to mediate MVB protein sorting. Vps4 uses energy from ATP hydrolysis to disassemble and recycle ESCRT-III for future MVB cargo sorting. To further determine whether the ESCRT pathway is involved in the release of Arc EVs, we overexpressed dominant negative Vps4 (DnVps4), including EGFP-Vps4A (E228Q) and EGFP-Vps4B(E235Q) (*SI Appendix*, Fig. S7*A*), and examined their effects on Arc puncta formation and EV secretion. To compensate for the effect of transfection of additional plasmids, we used a plasmid filler with no promoter (ΔCMV) as a control. Expression of dnVps4A and dnVps4B were verified by EGFP fluorescence. In these cells, HaloArc forms similar puncta as before. However, these puncta do not spatially overlap with dnVps4 (*SI Appendix*, Fig. S7*B*), and the number of HaloArc puncta per cell is consistent with that of control (*SI Appendix*, Fig. S7*C*). Furthermore, EV secretion is not affected by DnVps4 expression, and a substantial portion of EVs contain Arc (*SI Appendix*, Fig. S7*D*). Finally, EV concentration (*SI Appendix*, Fig. S7*E*) and sizes (*SI Appendix*, Fig. S7*F*) show similar values between Dn-Vps4 and control. These results suggest that the release of Arc-containing EVs is not inhibited by terminal disruption of the ESCRT pathway in HEK293T cells.

### RalA and RalB Double Knockout Alters the Morphology of HaloArc Clusters in Mammalian Cells.

Considering that purified Arc protein binds to PI3P and Arc puncta colocalizes with early endosomes, late endosomes, and MVB, and Arc puncta intensity progressively increases from early endosomes to MVB, we hypothesize that small oligomeric Arc could enrich at the early endosomes, and assemble capsids as Arc enters MVB (likely within the intraluminal vesicles), which undergoes membrane fusion with the plasma membrane to release EVs containing Arc capsid. To test this hypothesis, we manipulated the MVB biogenesis and compared phenotypic changes of Arc puncta. Ral GTPase is crucial for both MVB biogenesis and exocytosis (27, 41, 42). We, therefore, deleted endogenous RalA and RalB, two isoforms of Ral GTPase by CRISPR-knockout in HEK293T cells. RalA and RalB double knockout (DKO) was confirmed using qRT-PCR in a polyclonal population upon selection of the cells treated with the CRISPR-Cas9 machinery (*SI Appendix*, Fig. S8*A*). We further selected a monoclonal colony that had no expression for RalA and RalB (DKO) mRNA for further analysis (*SI Appendix*, Fig. S8*B*).

**Fig. 5.**
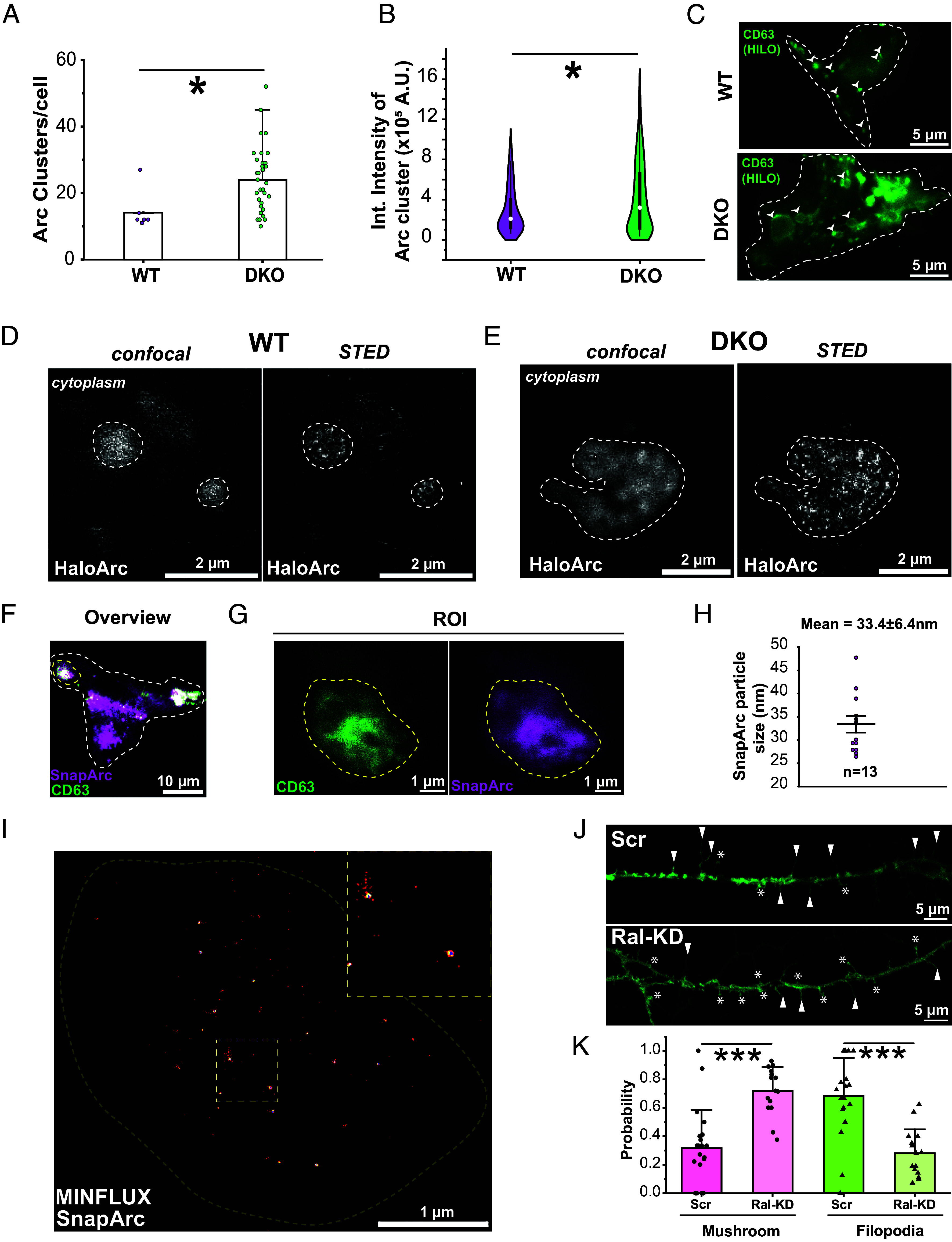
Decreased levels of RalA and RalB alters HaloArc trafficking and spine morphology. (*A*) Quantification of the number of HaloArc clusters in WT (pink) and DKO (green) HEK293T cells. (*B*) Quantification of integrated fluorescence intensity of HaloArc clusters in WT (pink) and DKO (green) HEK-293T cells. (*C*) A representative HILO image of WT HEK293T cell (*Top*) and DKO HEK293T cell (*Bottom*) expressing sfGFP-CD63. Representative images of HaloArc clusters inside the cytoplasm of wild-type (*D*) or DKO (*E*) HEK293T cells in confocal and STED modes. (*F*) A 2D-confocal image of a HEK293T cell expressing SnapArc (magenta, labeled with AF-647 SNAP-tag ligand) and sfGFP-CD63 (green). (*G*) Zoom-in view of an Arc-containing MVB (CD63). (*H*) MINFLUX imaging of SnapArc within the MVB in *G*. (*I*) Analysis of the size of SnapArc puncta resolved by MINFLUX. (*J*) Confocal imaging of cultured primary rat cortical neurons expressing membrane-associated EGFP cotransduced with either scrambled shRNA (*Top*) or RalA/B double knockdown shRNA (*Bottom*). Mushroom-like and filopodial spines are marked with asterisks and arrowheads, respectively. (*K*) Quantification of spine morphology in neurons treated with scrambled or RalA/B double knockdown shRNA (n = 21 dendrites for scrambled, n = 15 dendrites for knockdown condition) (Mann–Whitney test; *P < 0.05 and ***P < 0.001).

Similar to wild-type HEK293T cells, HaloArc forms puncta in DKO cells (*SI Appendix*, Fig. S8 *C* and *D*). These puncta colocalize with FYVE as in WT cells (*SI Appendix*, Fig. S8*E* and Movie S4). However, DKO cells show more Arc puncta per cell than wild-type cells (Fig. 5*A*, n = 9 cells for WT, n = 35 cells for DKO). In addition, the fluorescence intensity per puncta in DKO cells appears higher, with a larger average and integrated intensity than that of wild-type cells (Fig. 5*B* and *SI Appendix*, Fig. S9 *A* and *B*, n = 87 clusters for WT, n = 411 clusters for DKO). Additionally, puncta in DKO appeared to be larger (*SI Appendix*, Fig. S9*C*) and less circular (*SI Appendix*, Fig. S9*D*) than those in WT cells. These differences in puncta intensity and morphology imply erroneous MVB biogenesis in the DKO cells. In support of this idea, sfGFP-CD63 (MVB marker) positive vesicles were larger in DKO cells than those in wild-type cells (Fig. 5*C*). To better resolve intraluminal vesicles, we applied superresolution imaging. STED microscopy resolved multiple smaller puncta in both WT and DKO cells (Fig. 5 *D* and *E*). MINFLUX imaging of fixed HEK293T cells expressing SnapTag-Arc and sfGFP-CD63 resolved Arc clusters with an average diameter of 33.4 ± 6.4 nm (n = 13 particles, Fig. 5 *F*–*I*), which matches the size of the reconstituted Arc capsids measured by TEM (*SI Appendix*, Fig. S3*C*). These results suggest that Arc capsids could be encapsulated with intraluminal vesicles of MVB.

### RalA/RalB Double Knockdown in Cultured Rat Cortical Neurons Reduces the Percentage of Mature Dendritic Spines.

To determine the effects of RalA and RalB in neuronal cells, we compare the morphology of dendritic spines in wild-type and RalA/RalB double knockdown rat cortical neurons. Cells were treated with shRNA targeting RalA/RalB knockdown or scrambled shRNA. Knockdown was confirmed using qRT-PCR (*SI Appendix*, Fig. S9*E*). Cells were then infected with lentivirus encoding a plasma-membrane-associated EGFP and subjected to chemical depolarization at DIV 14 before live cell imaging on a confocal microscope. RalA/RalB double knockdown neurons show a higher percentage of mature mushroom spines (70%) compared to those treated by scrambled shRNA (40%) (Fig. 5 *J* and *K*), indicating that RalA and RalB can regulate the spine maturation in rat cortical neurons.

### RalA and RalB DKO Impairs Arc-Mediated Intercellular RNA Transfer.

**Fig. 6.**
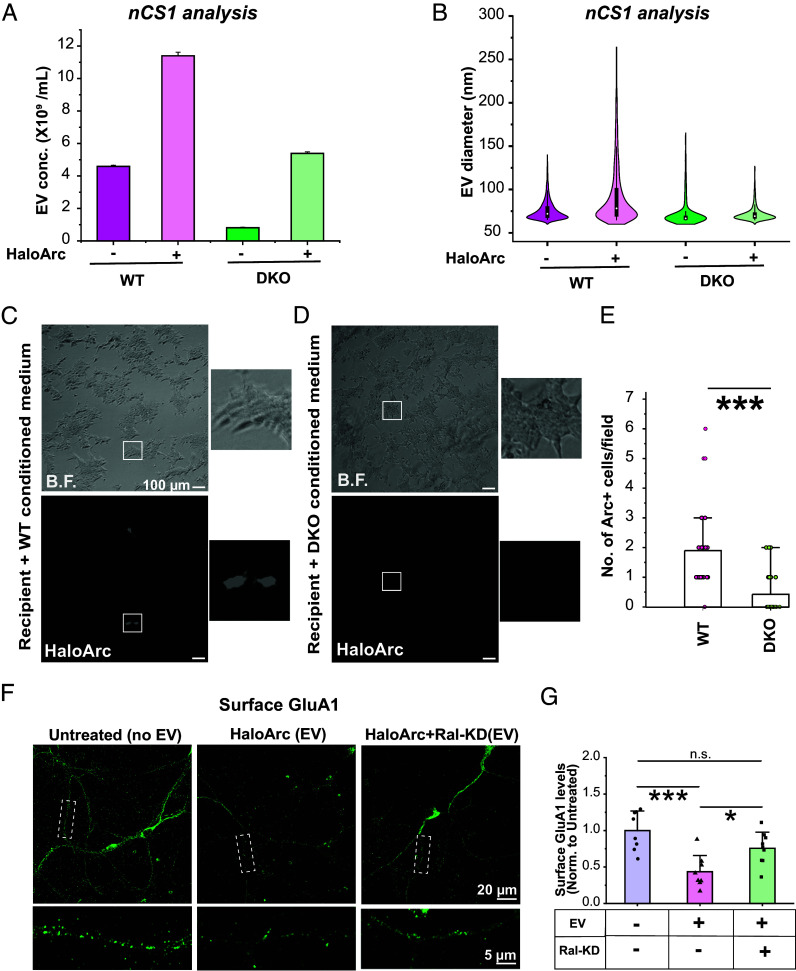
Effects of reduced levels of RalA and RalB on HaloArc-mediated intercellular RNA transfer and function in recipient cells. Comparison of EV concentration (*A*) and size (*B*) distribution of WT and DKO HEK293T cells with and without HaloArc expression. (*C*) Representative epifluorescence images of recipient cells treated with the conditioned medium from HaloArc-expressing wild-type cells. (*D*) Same as *C* except using the conditioned medium harvested from DKO cells. (Scale bars in *C* and *D*, 100 µm.) (*E*) Quantification of the RNA transfer efficiency from HaloArc-expressing wild-type (n = 29 images) or DKO cells (n = 27 images) (Mann–Whitney test; ***P < 0.001). (*F*) Immunocytochemistry of surface GluA1 subunit of AMPAR in untreated cultured mouse cortical neurons (*Left*) or those treated with EVs isolated from HaloArc-expressing wild-type (*Middle*) or RalA/B double knockdown (*Right*). (*G*) Quantification of the surface GluA1 level normalized to the average intensity from untreated neurons (n = 8 to 9 neurons each condition) (one-way ANOVA with the Tukey test *P < 0.05, ***P < 0.001, and n.s.: nonsignificant).

To determine whether defective MVB membrane fusion leads to impaired Arc capsid release, we used Spectradyne’s nCS1 Particle Analyzer to measure the concentration and size of EV harvested from the conditioned medium of wild-type and RalA/RalB DKO cells, which were transfected with the same amount of HaloArc. nCS1 measures both endogenous (Arc-free) and Arc-containing EVs extracted from the conditioned medium in HEK293T cells. Compared to nontransfected HEK293T cells, HaloArc expression in WT cells boosted EV release twofold, indicating Arc expression enhances EV secretion. In DKO cells, the basal level (Arc-free) EV concentration is significantly reduced, indicating a reduction of endogenous EV reduction. Although HaloArc expression still enhanced EV concentration in DKO cells, the absolute EV concentration is reduced by more than 50% compared to WT cells (Fig. 6*A*). HaloArc EVs in WT cells are larger than endogenous EVs, adding populations in the range of 100 to 250 nm in diameter. The larger EV size agreed with our STED images of the EVs (Fig. 1*D*). In DKO cells, this portion of larger EVs disappears (Fig. 6*B*).

We further confirmed that Arc-mediated intercellular RNA transfer is defective in the DKO cells using the donor-recipient RNA transfer assay. An identical amount of conditioned medium from WT and DKO donor cells expressing HaloArc was applied to two wells of equally plated HEK293T recipient cells. The transfection efficiency between WT and DKO donor cells is comparable. However, Arc expression in the recipient cells was significantly lower when incubated with the conditioned medium from DKO cells compared to that from wild-type cells, indicating defective intercellular Arc RNA transfer and protein expression in the recipient cells (Fig. 6 *C*–*E*). Cells positive for Arc fluorescence incubated with the DKO cell conditioned medium had a significantly lower fluorescence intensity compared to those treated with the WT cell conditioned medium (*SI Appendix*, Fig. S9*F*).

### Treatment of EVs Purified from RalA/RalB Double Knockdown Cells Fails to Reduce the Level of Surface GluA1 in Mouse Cortical Neurons.

To determine the functional outcomes of EVs in the recipient neurons, we purified EVs from the conditioned medium of cultured, HaloArc-expressing, wild-type, or RalA/RalB double knockdown rat cortical neurons. To stimulate EV secretion, we treated the culture with 50 mM of potassium chloride (KCl). Because Arc mediates the endocytosis of α-amino-3-hydroxy-5-methyl-4-isoxazolepropionic acid receptor (AMPAR), we compared the effects of EVs from wild-type and RalA/RalB double knockdown cells on the recipient neurons using immunocytochemical detection of surface GluA1 subunit of AMPARs. Fifty micrograms (50 μg) of EVs were incubated in DIV14 mouse cortical neurons for 4 h, followed by cell fixation and labeling of surface GluA1. Compared to the untreated condition, treatment of EVs purified from wild-type cells significantly reduced the level of surface GluA1 in the recipient neurons (Fig. 6 *F* and *G*). In contrast, neurons treated with EVs purified from RalA/RalB double knockdown cells showed a similar level of surface GluA1 as that of untreated neurons (Fig. 6 *F* and *G*).

## Discussion

**Fig. 7.**
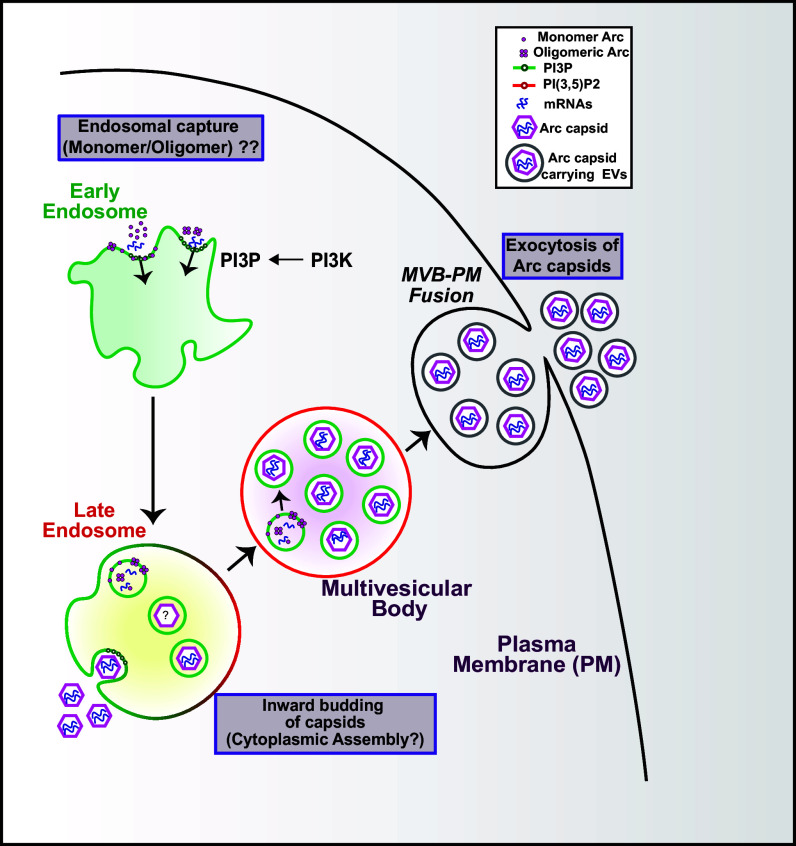
PI3P mediates Arc capsid trafficking and secretion through the endosome–MVB pathway. Arc protein is targeted to the limiting membrane of the early endosome through its interaction with PI3P (green), mediated by PI3K activity, and sorted through the axis of the late endosome and MVB. Endosomal PI3P could facilitate Arc oligomerization. Arc capsid assembly could occur either inside the cytoplasm or, more likely, within the intraluminal vesicles of MVB. Upon membrane fusion between MVB and the plasma membrane, EVs containing Arc are secreted.

In this work, we combined biochemical analysis, genetics, cell biological assay, live-cell, and superresolution imaging to define the intracellular axis of Arc capsid assembly and trafficking. Results suggest that the assembly and trafficking of Arc capsids is mediated through the endosomal–MVB pathway. Intriguingly, Arc capsid assembly and endosomal entry can be regulated by PI3P. Purified Arc proteins are associated preferably with PI3P. In mammalian cells, Arc colocalizes with FYVE, a PI3P marker. The high affinity between Arc and PI3P could recruit Arc proteins to endosomes or help with Arc oligomerization. Prolonged expression of FYVE reduces the average number of Arc puncta in cells, likely because of the competitive binding between FYVE and Arc to PI3P. Within the early endosomes, the protein undergoes sorting processes, likely involving specific protein–protein interactions or recognition motifs. This sorting directs the protein-containing vesicles toward late endosomes. Upon endosomal maturation, Arc capsids are packaged into individual intraluminal vesicles within MVB. As the MVBs mature, they migrate toward the cell periphery. Fusion between MVB and the plasma membrane releases Arc capsids as EVs (Fig. 7). RalA and RalB DKO in HEK293T cells results in the accumulation of Arc puncta inside MVB, indicating their role in regulating the secretion of Arc-containing EVs. Double knockdown of RalA and RalB in cultured rat cortical neurons results in an increase in the mature spines upon chemical depolarization, consistent with the findings that RalA mediates the endocytosis of AMPAR (43). When incubated with cultured mouse cortical neurons, EVs purified from HaloArc-expressing neurons reduce the level of surface GluA1 subunit of AMPAR, consistent with a recent work (44). However, such reduction did not occur when EVs were purified from RalA/B double knockdown neurons.

It is unclear whether Arc enters endosomes as a complete capsid or incomplete assembly. We speculate that full capsid assembly is more likely to occur after endosomal entry due to the enrichment of Arc proteins. Live-cell imaging shows Arc puncta intensity is lower when colocalized with Rab5-positive early endosomes compared to Rab7- and CD63-positive MVBs. ILV entry of Arc may involve the endosomal sorting complex required for traffic (ESCRT) proteins (45). Conventionally, ESCRT components 0, I, and II help in the clustering of cargoes, curving the membrane and inducing their entry into the intraluminal vesicles inside the MVBs. ILV entry can also involve ESCRT-III, ALIX, and syntenin (46).

Trafficking through the endosomal–MVB pathway implies Arc capsids utilize distinct sorting signals to differentiate from other ILVs, which typically undergo degradation when MVB fuses with the lysosome. We speculate that the Rab family proteins, such as Rab11, that control endosome-to-plasma membrane recycling, could mediate Arc sorting. It has been suggested that Rab11 could promote cargo loading into the EVs or protect cargoes from lysosomal degradation. Recycling endosomes can populate neuronal EV precursor populations (47). Specifically, the loss of Rab11 reduces Arc levels in *Drosophila* EVs (15).

PI3P is enriched in the limiting membrane of early endosomes. The high affinity of Arc to PI3P could facilitate the initial enrichment and recruitment of Arc to the early endosomal membrane. It is currently unclear which domain of Arc protein interacts with PI3P and whether the binding to endosomal membrane is posttranslationally regulated or constitutive. Stahelin and coworkers previously demonstrated that the PI3P interacting domain, FYVE, can bend the endosomal membrane, thereby increasing its residence time with the endosomes (48). It is conceivable that Arc binding also induces local changes in the endosomal membrane, which allows the entry of oligomeric Arc into the lumen of the endosomes. PI3K activity is necessary for Arc association with the endosomal membrane, consistent with previous findings that MVB morphogenesis requires PI3K activity (49).

PI3P could also play a role in mediating Arc capsid assembly. Biochemical analysis revealed that mammalian Arc consists of a positively charged N-terminal (NT) domain and a negatively charged C-terminal (CT) domain, separated by a flexible linker (50). In vitro experiments showed that Arc capsid assembly is mediated through the N-terminal domain, particularly the second helical structure, Coil-2 (51). Capsid assembly may start with Arc oligomerization on PI3P-enriched early endosomes. Such Arc oligomerization in the reducing cytosolic environment is possible because previous work showed that the five cysteine residues (Cys34, Cys94, Cys96, Cys98, and Cys159) did not affect the Arc–Arc interaction, suggesting that oligomerization does not require disulfide bond formation (51). Compared to monomeric Arc, Arc oligomers bind to PI3P with higher avidity, which could gate the initiation of precise Arc capsid assembly by ensuring sufficient capsid building blocks. Oligomeric Arc could be invaginated from the limiting membrane of MVB and become intraluminal vesicles, where full capsid can be assembled as the endosomes mature.

The assembly and trafficking of capsids of Arc and HIV Gag protein present intriguing parallels and distinctions, offering insights into commonalities and unique mechanisms underlying these processes within biological systems. Similar to other viral Gag proteins, Arc proteins self-assemble into capsids in the presence of phosphate or nucleic acids. Both Arc and HIV rely on multimerization for capsid assembly. Arc, a neuronal protein, forms virus-like capsids through self-oligomerization, a process akin to the assembly of retroviral capsids. This self-assembly ability mirrors the structural arrangement observed in the assembly of HIV capsids, where the Gag polyprotein undergoes multimerization to form the viral capsid. Both Arc and HIV capsid shells serve as a protective convoy vehicle that protects the encapsulated genetic materials.

Phospholipids seem to play a crucial role in the assembly of HIV and Arc capsids in mammalian cells. It is known that HIV capsid proteins reconstitute capsid-like structures in vitro but form only 25 to 30 nm in size unless mixed with inositol phosphates like IP5 and PIP3, which restores the 100 to 120 nm HIV capsids comparable to their sizes assembled in cells (52). In addition, the HIV gag specifically restricts PI(4,5)P2 and cholesterol on the plasma membrane for its assembly (53). Indeed, Linwasswe and coauthors showed that the HIV-gag assembly site is regulated by the subcellular distribution of cholesterol (54). In this work, we found that phospholipids regulate Arc capsid assembly in mammalian cells. However, Arc has a higher affinity to PI3P, an endosome-specific phospholipid.

In contrast to the asymmetric cone formed by HIV capsids, Arc forms a spherical capsid with a diameter of 30 nm (Figs. 3*D* and 6 *H* and *I*), consistent with previous findings (14). This difference might result from distinct monomer interface and oligomeric units formed by HIV capsid protein and Arc. HIV can form slightly different pentameric and hexameric assemblies, both serving as building blocks that tile capsid surfaces. Another difference is that HIV capsids are commonly believed to be released at the plasma membrane, whereas we found that Arc capsids secrete through the endosomal–MVB signaling axis. However, HIV capsid release might be cell-type specific. Ono A. et al. previously compared HIV assembly of MA mutants in different cell types and discovered that HIV can also utilize the MVB pathway for its assembly and egress upon removal of plasma membrane targeting basic MA domain residues. In fact, monocyte-derived macrophages targeted the WT gag to the intracellular vesicles, which was confirmed by colocalization with CD63 and Rab7 (55). The MVB pathway utilization has also been the murine leukemia virus (MLV) replication (56). Interestingly, terminal perturbation of the ESCRT pathway does not affect Arc capsid assembly and release into the extracellular spaces, which is consistent with the paradigm that lipid-mediated EV biogenesis could be ESCRT-independent (57).

Synaptic development is an important function of the neuronal IEGs and corresponds to their link with various neurological conditions when dysregulated. Arc is different from other IEGs in that Arc is not a transcription factor but rather a major signaling hub at distinct subcellular compartments such as synapses (58–60), axons (61), or the nucleus for transcriptional regulation (62) and chromatin modification (63). The recently discovered Arc function in regulating intercellular RNA transfer requires a stable infrastructure where Arc can go through intracellular assembly into a capsid and intercellular delivery of genetic materials. On the one hand, genetic materials encapsulated in the capsids sample the intracellular state at the moment of release. On the other hand, these materials could define the Arc’s remote functionality in the recipient neurons yet to be discovered.

The involvement of PIP lipids in Arc neuronal intercellular communication is intriguing but makes sense, considering that PIP lipids have a major role in neuronal function, particularly in modulating synaptic strength. PI3P is an important signaling lipid and is converted to its important metabolite PI(3,5)P2 using PIKfyve (38, 64). PI(3,5)P2 levels bidirectionally control the synaptic strength, with high PI(3,5)P2 causing synaptic pruning and low PI(3,5)P2 levels causing synapse strengthening. PI(3,5)P2 repression also reduces AMPAR endocytosis, a process also directly controlled by Arc protein (65). Interestingly, the PI3P-producing enzyme Vps34 is highly expressed in the central nervous system neurons and is highly localized at the dendritic spines. Loss of Vps34 is associated with loss of dendritic spines and gliosis and neurodegeneration in aged mice (66), supporting the idea that higher PI3P levels might be required during synaptic strengthening, such as long-term potentiation (LTP). The neuronal function might depend on the PI3P: PI(3,5)P2 ratio as several genes related to the synthesis of PI(3,5)P2 from PI3P have been implicated in critical neurological conditions like epilepsy, neuropathy, and neurodegeneration. As an early endosome and MVB marker, PI3P regulates the recruitment of PH-, PX-, and FYVE domain-containing proteins to MVB compartments (67). Initial membrane attachment of proteins can induce dimerization and further penetration of hydrophobic amino acids into the intracellular membrane (48, 67).

We noted that Arc puncta populated across the dendrites in cortical neurons (Fig. 2*H*). In supporting this idea, electron microscopy also resolved immunogold-labeled Arc in the dendritic spines (68). As shown in this work, the release of Arc capsids can go through the MVB pathway, mediated by Ral GTPases. Indeed, neuronal MVBs have been implicated in various aspects of neuronal system regulation via cellular material exchange (69, 70). Electron microscopy has revealed that the dendritic shafts of the neurons are enriched with MVBs, with a fraction of spines of the glutamatergic synapses also carrying some MVBs (71, 72). Strikingly, somato-dendritic compartments carry about 50 times more MVBs than axons (73). MVBs migrate toward synapses in response to synaptic activity and learning behavior (e.g., water maze) in rats (74). Chronic stress, on the other hand, reduces the association of MVBs with PSD (74). The dendritic membrane undergoing membrane fusion with MVB could be a potential site for Arc capsid release. However, a direct visualization of such an event is yet to be elucidated.

Interestingly, a recent study has shown that another synaptic protein, gephyrin, found at the inhibitory synapses, also clusters in response to PI3P in cultured hippocampal neurons, thereby determining the strength of inhibitory GABAergic synapses (39). In line with this idea, defective PI3P synthesis could lead to improper Arc release and overaccumulation of Arc at the dendrites, causing silencing and even loss of the spines. This loss remains permanent and could lead to progressive degeneration in the brain (66). Evidently, AD patients have a PI3P deficiency, and PI3P levels are genetically linked with ALS and PD (75). Understanding the cellular machinery orchestrating Arc capsid assembly and release promises to shed light on the commonality and distinction of trafficking pathways between structurally resembled capsid proteins, as well as intercellular communication between distant neurons.

## Materials and Methods

### Donor–Recipient Transfer Assay.

HaloArc transfer was assayed by transferring conditioned medium from HaloArc-transfected or untransfected cells (donor) to untransfected cells (recipient). Briefly, donor cells were cultured on a 10-cm dish and transfected with 10 μg HaloArc plasmid. Cells were washed twice with DPBS to remove any excess plasmid-PEI aggregates after 5 h of transfection, and the fresh growth medium was added. The conditioned cell medium from transfected donor cells was harvested 24 h later and spun at 500×*g* for 5 min to remove any cell debris. Cleared supernatant was then added to the recipient cells and incubated for another 24 h. Recipient cells were stained after donor media treatment across the entire well.

### PIP-Strip Lipid Overlay Assay.

A 0.5 µL purified protein was spotted and dried out completely in the corner of the PIP strip (Echelon Bio.) to serve as the positive control. Later, the strip was incubated in a blocking solution (5% milk in TBS, pH 7.2) on a rocking platform for 1 h at RT. Protein was diluted in fresh blocking buffer and incubated with the strip (2 h, RT), washed, and probed with the primary Arc antibody (Synaptic Systems) (1:1,000, 1 h, RT), followed by incubation with HRP-conjugated secondary antibody (1:10,000, 1 h, RT) on a rocking platform. The strip was washed thrice and then imaged for chemiluminescence using freshly prepared Clarity ECL western substrate (Biorad).

### Particle Tracking and Diffusion Coefficient Calculation.

Particles were tracked from the HILO-acquired videos using the FIJI plugin TrackMate (76) (Software S1), which calculated the particle diffusion coefficient using the co-variance estimation (CVE) method (77, 78).

### Data Analysis, Statistics, and Data Visualization.

HaloArc cluster parameters were calculated using the “Analyze Particles” feature of FIJI. Images were initially convolved using the *Convolve* filter in FIJI for background subtraction and feature enhancement. Particles were then analyzed, and all the measured parameters were exported to OriginPro 2022 and plotted into histograms and violin plots. Datasets were compared using the Mann–Whitney *U* or the Kruskal–Wallis ANOVA nonparametric test as it does not assume the normal distribution of the datasets.

## Supplementary Material

Appendix 01 (PDF)

Dataset S01 (CSV)

Dataset S02 (CSV)

Dataset S03 (CSV)

Movie S1. **Representative time-stamped HILO imaging of a HEK293T cell expressing HaloArc showing intracellular HaloArc clusters into slow diffusing puncta.** Cells are labeled with 200nM JF549 HaloTag ligand. Warmer colors indicate higher intensities. Related to Fig. 2A.

Movie S2. **Representative time-stamped HILO imaging of a HEK293T cell expressing HaloTag showing fast diffusing intracellular Halotag molecules.** Cells are labeled with 200nM JF549 HaloTag ligand. Warmer colors indicate higher intensities. Related to Fig. 2B.

Movie S3. **Representative time-stamped HILO imaging of a WT HEK293T cell co-expressing HaloArc (magenta) and EGFP-2xFYVE (green) showing colocalization of Arc clusters with PI3P carrying vesicles.** Cells are labeled with 200nM JF549 HaloTag ligand. Related to Fig. 3C.

Movie S4. **Representative time-stamped HILO imaging of a DKO HEK293T cell co-expressing HaloArc (magenta) and EGFP-2xFYVE (green) showing colocalization of Arc clusters with PI3P carrying vesicles.** Cells are labeled with 200 nM JF549 HaloTag ligand. Related to Fig. S8E.

Movie S5. **Representative time-stamped HILO imaging of a HEK293T cell co-expressing HaloArc (magenta) and EGFP-Rab5 (green) showing localization of Arc clusters with early endosomes.** Cells are labeled with 200nM JF549 HaloTag ligand. Related to Fig. 4A.

Movie S6. **Representative time-stamped HILO imaging of a HEK293T cell co-expressing HaloArc (magenta) and sfGFP-CD63 (green) showing localization of Arc clusters with MVBs.** Cells are labeled with 200nM JF549 HaloTag ligand. Related to Fig. 4B.

Movie S7. **Representative time-stamped HILO imaging of a HEK293T cell co-expressing HaloArc (magenta) and EGFP-Rab7 (green) showing localization of Arc clusters with late endosomes and MVBs.** Cells are labeled with 200nM JF549 HaloTag ligand. Related to Fig. 4C.

Movie S8. **Representative time-stamped HILO imaging of a HEK293T cell co-expressing sfGFP-Arc (green) and mCherry-TSG101 (magenta) showing Arc clusters also localize with ESCRT protein TSG101.** Cells are labeled with 200nM JF549 HaloTag ligand. Related to Fig. S6A–B.

Movie S9. **Representative time-stamped HILO imaging of a HEK293T cell co-expressing MVB marker mCherry-TSG101 (magenta) and EGFP-Rab7 (green) showing late endosomes and MVBs colocalize.** Cells are labeled with 200nM JF549 HaloTag ligand. Related to Fig. S6C–D.

## Data Availability

Software data have been deposited in Github (https://github.com/kritikamehta1794/Diffusioncoefficient_CVE.git) (79). All other data are included in the manuscript and/or supporting information.
