# Supplementary material for: Phosphatidylinositol-3-phosphate mediates Arc capsid secretion through the multivesicular body pathway
Source: Proc Natl Acad Sci U S A. 2024 Aug 23;121(35):e2322422121. doi: 10.1073/pnas.2322422121 (PMC11363301; doi:10.1073/pnas.2322422121)
Supplement: Supplementary file 1 — Appendix 01 (PDF) [file pnas.2322422121.sapp.pdf]

PNAS

Supporting Information

Phosphatidylinositol 3-phosphate mediates Arc capsid secretion through the multivesicular body pathway

Kritika Mehta[a,b], Henry Yentsch[a], Jungbin Lee[a], Yeeun Yook[c], Kwan Young Lee[c], Tianyu Terry Gao[a], Nien-Pei Tsai[c], Kai Zhang[a,b,d,e,f,g,1]

[a] Department of Biochemistry, University of Illinois Urbana-Champaign, Urbana, IL 61801

[b] NSF Science and Technology Center for Quantitative Biology, University of Illinois Urbana-Champaign, Urbana, IL 61801

[c]Department of Molecular and Integrative Physiology, University of Illinois Urbana-Champaign, Urbana, IL 61801

[d] Center for Biophysics and Quantitative Biology, College of Liberal Arts and Sciences, University of Illinois Urbana-Champaign, Urbana, IL 61801

[e]Neuroscience Program, College of Liberal Arts and Sciences, University of Illinois Urbana-Champaign, Urbana, IL 61801

[f]Beckman Institute for Advanced Science and Technology, University of Illinois Urbana-Champaign, Urbana, IL 61801

[g]Cancer Center at Illinois, University of Illinois Urbana-Champaign, Urbana, IL 61801

[1]To whom correspondence may be addressed. Email: kaizkaiz@illinois.edu.

This PDF file includes:

Supporting text
Figures S1 to S9
Tables S1 to S3
Legends for Movies S1 to S9
Legends for Datasets S1 to S3

Other supporting materials for this manuscript include the following:

Movies S1 to S9
Datasets S1 to S3
Software S1

Supplementary Methods

Plasmid construction and lentivirus production

The HaloArc sequence was cloned from pRK5-mHalo-Arc plasmid, a gift from Prof. Jason Shephard, University of Utah. The HaloArc sequence was inserted into the multiple cloning site (MCS) of an empty pCS2+ vector (NovoPro) using InFusion cloning after double digestion with FD-EcoRI (Thermo) and FD-NotI (Thermo). HaloTag was replaced with sfGFP using InFusion (Takara) assembly by cutting out the HaloTag using FD-PacI (Thermo) and FD-BcuI (Thermo). HaloTag and sfGFP were independently amplified from the HaloArc and sfGFP-Arc plasmids, respectively and cloned into an empty pCS2+ vector using InFusion assembly. For lentivirus transfer plasmids, the HaloArc sequence was inserted into a backbone after double digestion with FD-NheI (Thermo.) and FD-EcoRI (Thermo.) using InFusion cloning (Takara). For neuron knockdown, two shRNA encoding sequences (https://sirna.wi.mit.edu/) were inserted into FD-EcoRI and FD-BshTI digested pLKO.1 vector using a strategy similar to that of KO plasmids (see above). Low-passage (< p10) HEK293T cells were plated in growth media without penicillin or streptomycin and incubated overnight. A transfection solution of 3:2:1 of the plasmid of interest, psPAX2 (packaging), and pMD2.G (envelope) plasmids diluted in Opti-MEM was created, along with a 3:1 dilution of PEImax diluted in Opti-MEM. After 5 minutes, the two solutions were combined, incubated for 30 minutes at room temperature, and added dropwise to cells. After exactly 16 hours post-transfection, the media was changed to complete growth media. After 24 hours of incubation, the media was harvested and stored at 4 °C and replaced with fresh growth media. After another 24 hours of incubation, the media was harvested again and combined with the previously stored media. The media was centrifuged for 5 minutes at 500 g and filtered through a 0.45 µm syringe filter (Millipore). The media was concentrated with a UV-sterilized Amicon ultra-15 100 kDa centrifugal filter until the solution was concentrated by 30 times its original volume. Aliquots of this concentrated media were made and stored at -80 °C. The plasmid, primers, and reagent information are shown in **Tables S1, S2, and S3**.

Protein purification and characterization

Arc protein was overexpressed using the pGEX-6p1-rArc construct, a gift from Prof. Jason D. Shepherd, University of Utah. Briefly, house-made chemically competent BL21(DE3) cells were transformed with the plasmid. A starter bacterial culture was grown overnight at 37 °C in Luria broth LB (Fisher) supplemented with ampicillin (Fisher). Starter cultures were used to inoculate large-scale 500 mL cultures of LB. Once the culture was grown to OD_{600} of 0.6-0.8 at 37 °C at 150 rpm, the culture was moved to a 19 °C incubator set at 150 rpm for 16-20 h. Cells were pelleted at 5000 g for 15 min at 4 °C and cell pellets were resuspended in 30 mL lysis buffer (500 mM NaCl, 50 mM Tris, 5% glycerol, 1 mM Dithiothreitol DTT, 1 mM Phenylmethylsulfonyl fluoride PMSF, pH 8.0 at room temperature (RT)). Resuspended cells were flash-frozen in liquid nitrogen and stored at -80 °C overnight. The next day, frozen pellets were thawed quickly at 37 °C and brought to a final volume of 1 g pellet:10 mL lysis buffer. Lysates were then sonicated for 5 minutes with 2.5 sec on, 2.5 sec off at 40% amplitude and pelleted for 45 min at 21,000 g. Cleared supernatants were then passed through a 0.45 µm filter and incubated with pre-equilibrated Glutathione Sepharose 4B affinity resin (Cytiva) in a gravity flow column overnight at 4 °C on a rolling platform. Bound protein was then washed twice with two column volumes of lysis buffer, re-equilibrated with cleavage buffer (150 mM NaCl, 50 mM Tris, 1 mM Ethylenediamine tetra-acetic acid EDTA, 1 mM DTT, pH 7.2 at RT), and cleaved on-resin overnight at 4 °C with 100uL PreScission Protease (GE). Elution was collected the next day with 5mL elution buffer (50mM Tris, 10mM reduced L-glutathione, pH 8.0). Cleaved proteins were run on an S200 size exclusion

column (Cytiva) to separate the cleaved protein and peak fractions were pooled. Protein was concentrated and buffer exchanged in TBS buffer (50 mM Tris, 150 mM NaCl, 1 mM DTT, pH 7.4). Average yields for purified proteins were ~10 mg per 500 mL of cell culture.

Cell culture and transfection

HEK293T cells were cultured in Dulbecco's Modified Eagle Media (DMEM, Corning) supplemented with 100 units/mL Penicillin, 0.1 mg/mL Streptomycin, 2 mM L-Glutamine and 10% fetal bovine serum (Sigma Aldrich). The transfection mix was prepared by diluting plasmid DNA in fresh DMEM without supplements. PEImax (PolyScience) was used to carry out transfection and was added as per the vendor's instructions. Media was changed back to growth media 3-5 hours post-transfection. For Wortmannin treatment, cells transfected with HaloArc were incubated with 5 µM drug (or an equal volume of DMSO for control) overnight to allow consequent PI3K inhibition.

Primary Neuronal Culture and Lentiviral transduction

Coverslips were coated with 100 µg/mL Poly-D-Lysine (PDL) diluted in Borate buffer. The next day, PDL on the coverslips was aspirated out and washed twice in autoclaved Mili-Q water. Coverslips were left to dry inside the laminar airflow under UV for about 20 minutes until they were ready for plating. Brains from E18-E19 rat embryos were placed in a large Petri dish containing 1× slice dissection solution (SLDS) at 4˚C. Cortices were dissected out of the brains, and about 2 mL protease enzyme solution was added to the cortices and incubated inside an incubator at 37 °C for 30 minutes for dissociation. The enzyme solution is removed gently using a pipet, and a 3 mL plating medium is added into a conical tube containing the cortices. Tissues are dissociated into single cells using polished glass pipets. Cells are then counted on a hemocytometer, and about 500,000 neurons are plated per coverslip in a plating medium. Media was changed to neurobasal (NB) media after 3 hours of plating. Half of the NB media was changed every 3 days to avoid loss of growth factors.

Neuron transduction was performed between DIV6 and 7. Briefly, half of the NB media was removed, and an appropriate content of the virus was added to the neurons. After 3 hours of incubation, the NB media containing the virus was removed and stored NB was added back to the transduced neurons with equal volume of fresh NB. Neurons were maintained normally for 6-12 days to allow expression. Transduced cells to be imaged were labeled with 200 nM of JF-549 HaloTag ligand and were kept in ACSF during the destaining step. Labeled neurons were fixed with 4% paraformaldehyde, immunolabeled for MAP2 and mounted onto the glass slides using the ProLong Gold antifade reagent with DAPI (Thermo.). All information regarding the reagents has been provided in **Table S3**.

CRISPR knockout and monoclonal selection

A total of 4 gRNAs were designed for both hRalA and hRalB genes using CHOPCHOP (https://chopchop.cbu.uib.no/). All knockout plasmids were directly transfected into the cells using PEImax. Exactly 16 hours after transfection, cells were recovered in growth media supplemented with 1.2 µg/mL puromycin. Cells were cultured in selection media for another 2 weeks. Monoclonal selection was performed with selected cells in a 10-cm dish. Colonies started to appear in about 10 days and were picked and transferred to individual wells of a 24-well plate. Double knockout was confirmed from reverse transcribed cellular RNA against hRalA and hRalB specific primers.

Fluorescence labeling of HaloArc and Immunofluorescence

Transfected cells were stained with JF-549 tagged HaloTag ligand (Promega) and Vybrant DiO staining solution (Invitrogen) in phenol red-free DMEM for about 15-20 min at 37 °C at a final concentration of 200 nM and 1 µM respectively. For HEK293T labeling, cells were washed twice with Dulbecco's phosphate-buffered saline with Calcium and Magnesium (Corning) to minimize cell detachment. For primary neuron labeling, neurons cultured on coverslips were stained in the NB media. Neurons were washed and destained in artificial cerebrospinal fluid (aCSF) media with or without high KCl (50 mM) for 1 hour. Neurons were then fixed in 4% paraformaldehyde and 3% sucrose in PBS for 15 minutes on ice. After fixation, neurons were permeabilized in 0.2% TritonX-100 in PBS for 10 minutes. Neurons were blocked in 1% BSA in PBS for 30 minutes at RT. Blocked neurons were incubated with primary MAP2 antibody diluted 1:2000 in blocking buffer for 16 hours at 4 °C . After washing with PBS, cells were incubated with 1:1000 dilution of Alexa-647 labeled secondary antibody for 2 hours at RT. Coverslips were mounted onto glass slides using the Prolong Gold Antifade mounting medium with DAPI (Thermo.) and cured overnight in the dark.

Western Blot

Transfected cells were lysed 24 hours post-transfection using cold lysis buffer (1× RIPA, 1× protease inhibitor cocktail). Cell lysates were centrifuged at 17,000 g for 10 minutes at 4 °C. Protein in the supernatant was quantified on a plate reader using the Bradford assay (Sigma Aldrich). All samples were prepared in loading dye (NU-PAGE dye + 5mM BME) and boiled at 99 °C for 5 minutes. Equal amounts of denatured lysates were loaded on a 12% SDS gel and ran with SDS running buffer (Biorad) for 90 minutes at 100 V. An overnight transfer was performed to capture proteins onto PVDF membrane (Biorad) with transfer buffer (25 mM Tris-base, 192 mM Glycine, 10% Methanol). The membrane was blocked in a blocking buffer (5% milk in Tris-base, 1%Tween-20; called TBST) and was incubated with primary antibodies overnight at 4ºC. After washing with TBST, the membrane was treated with HRP-conjugated secondary antibody and later stained and imaged for chemiluminescence using freshly prepared Clarity ECL substrate (Biorad). For testing PI3K inhibition, serum-deprived HEK293T cells were incubated with different doses of Wortmannin for 6 hours, followed by stimulation of 10% FBS growth media for 15 minutes. Cells were then lysed for Western blot analysis.

Reverse transcription PCR

Total cellular RNA was extracted using the GeneJet RNA extraction kit (Thermo). A total of 1 µg of cDNA was prepared using the M-MulV reverse transcriptase (NEB) as per vendor instructions. A total of 10 ng cDNA was used to run qPCR using the SYBR green master mix (Applied Biosystems). All gene-specific primer sequences have been provided in **Table S2**. The reactions were run on an Applied Biosystems quantitative PCR machine, and all the acquired Ct values were analyzed. In all the samples, the *36B4* gene served as the internal control gene. To confirm the CRISPR gene knockout for RalA and RalB in the monoclonal cells, we ran a regular PCR using the Taq polymerase master mix (Promega) using RalA/B-specific primers. Gene knockout was thereby confirmed by gel electrophoresis.

Epi-illumination Microscopy

Cells were finally imaged in serum and phenol red-free DMEM (Gibco) at 40× air and 100× oil objective using an epi-illumination inverted fluorescence microscope (Leica DMI8) equipped with

both the objectives and a light-emitting diode illuminator (SOLA SE II 365). JF-549 fluorescence was detected using the mCherry filter cube (Leica, excitation filter 560/40, dichroic mirror 585, and emission filter 592/668) with an exposure time of 500 ms for JF549 (red), 200 ms for Alexa 647(far red), 200 ms for DAPI (blue) and 50 ms for DiO (green), and 2×2 binning.

HILO fluorescence imaging

Transfected cells cultured on coverslips were stained with JF-549 HaloTag ligand and were imaged under a custom-built HILO microscope. Cells were tracked for 20 s using an integration time of 100 ms in HILO mode. A continuous wavelength (561 nm, Spectral physics) laser was used as the light source. An inverted microscope (IX73, Olympus) equipped with a 100× oil immersion TIRF objective (Olympus, PlanApo, 100×, NA 1.49, oil immersion) was used. The laser beam was then expanded, collimated to about 35 mm, and directed into the microscope by a lens (400 mm focal length, Thorlabs LA1725A). The incident light was directed through the objective via an exciter (FF01-482/ 563-25) and a dual-band dichroic filter (Di01-R488/561−25 × 36). The cellular fluorescence was collected by the same objective, passing an emitter (FF01-523/610-25) and captured by an electron multiplying charge-coupled device (EMCCD) camera (iXon U797, Andor Technology). Arc clusters were detected as bright intracellular clusters. HaloTag transfected cells, serving as a negative control, were labeled and tracked with an integration time of 10 ms achieved by reducing the size of the field to 256×256 pixels.

For two-color imaging in the HILO mode, the incoming fluorescence from the microscope was split into red and green light using the Opto-Split II (Cairn Research) setup installed between the camera and the light path (**Fig. S3A**). Fluorescence was separated by two filter sets (FF03-482/563-25 for green emission and FF02-523/610-25 for red emission. Emitted light was split using a 575LP (Di R488/561). Co-transfected cells were prepared and illuminated concurrently with 488 and 561 nm lasers. The split images were visualized on a split screen on a computer. Tracking was performed with an integration time of 100 ms for 20 s. Images were later cropped and merged in FIJI (https://fiji.sc/). All videos were analyzed for colocalization, intensity and diffusion coefficient calculations (see below).

Super-resolution imaging (STEDYCON and MINFLUX)

All super-resolution imaging was performed at the core facilities at the Carl R. Woese Institute of Genome Biology, UIUC. For STED imaging, cells and EV samples were labeled with 200 nM JF646 HaloTag ligand. EVs were co-stained with 1 µM DiO to locate them. The samples were fixed and mounted onto glass slides using a DAPI-free mount. Samples were initially imaged in confocal mode, and then a selected ROI was run in STED mode using the Abberior STEDYCON setup. For MINFLUX imaging, cells were co-transfected with SnapArc and sfGFP-CD63 constructs, fixed for 30 s in 2.4% paraformaldehyde, and permeabilized with 0.4% TritonX-100 for 3 min and fixed again in 2.4% paraformaldehyde for 30 min. Cells were quenched with 100 mM NH_4Cl prepared in PBS. After washing twice with PBS, cells were blocked with Image-IT signal enhancer (Thermo.) for another 30 min. Alexa-647 Snap dye (NEB) was diluted in the staining solution (1 mM DTT+0.5% BSA in PBS) to a final concentration of 100 mM and added to cells for 50 min. Cells were washed 2-3 times in PBS. Shortly before imaging, coverslips were treated with gold beads (BBI solutions) for 10 min. Coverslips were washed multiple times and were inverted onto a cavity slide containing imaging buffer (50 mM Tris-HCl, 10 mM NaCl, 10% glucose, 64 µg/mL catalase, 0.4 mg/mL glucose-oxidase,10 mM cysteamine, pH=8.0) and sealed with a two-component silicone glue (Picodent, Twinsil). Cells were first imaged using the 488 nm and 640 nm lasers in confocal mode to find a region of interest. The region of interest was then

cropped from the whole image and captured in a new window with a pixel size of 20 nm. This selected ROI was used to carry out MINFLUX imaging using the 640 MINFLUX laser. Data was collected for about 30 min, and a 405 nm laser was used to re-activate the fluorophores. Images in confocal (for CD63) and MINFLUX-mode (SnapArc) were overlayed after resizing in FIJI.

Extracellular vesicle isolation and quantification

HEK293T cells were cultured in standard growth media for 24 hours post-transfection before media harvest. EV-depleted growth media was prepared using a 100 kDa cut-off ultrafiltration filter (Amicon) by centrifugation of FBS at 3,000 g for 55 minutes before media preparation. For nCS1 experiments, transfected cells were cultured in EV-depleted growth media for an additional 24 hours before media harvest. The Capturem EV isolation mini kit (Takara) was used following the manufacturer's instructions. Briefly, the conditioned media was spun at 500 g for 5 minutes and then at 20,000 g for 30 minutes. The collected supernatant was loaded onto the pre-clearing columns to remove larger particles larger than 800 nm. The cleared media was then concentrated using a 100 kDa cut-off ultrafiltration filter by spinning at 3000 g until the final volume was reduced to about ~1mL. The concentrated media was then loaded onto the purification column, and EVs were eluted in the elution buffer. Neuronal EVs were isolated from the neuro basal media from DIV15 rat cortical neurons following lentiviral treatment. The conditioned neuronal media was filtered using the 0.8μm syringe filter (Sartorius) and then processed further using the Qaigen ExoEasy Maxi kit according to the manufacturer's instructions. After purification, the eluted EVs underwent buffer exchange into 1X PBS prior to their use in functional assays on mouse neurons.

The concentration and size of EV was measured using Spectradyne nCS1. Eluted EVs were buffer exchanged with freshly prepared, 0.22 μm-filtered 0.1%-PBST (pH=7.4). A 5 μL sample was applied to the Spectradyne C-400 cartridges (diameter range 65-400 nm). The samples were run on the nCS1 instrument until at least 5000 particles were analyzed. Peak data was filtered according to the guidelines in the Spectradyne nCS1 manual. False-positive particles are characterized as having transit times >100 μs, diameters outside of 65 to 400 nm, a signal-to-noise ratio of <15, or a peak symmetry value outside of 0.2 to 4.0. Data and statistical analysis were performed using OriginPro (2021b). Two-color fluorescence imaging of EV was performed by staining concentrated EVs with 5 μM DiO cell staining solution (Invitrogen) and 100 nM JF-549 HaloTag ligand (Promega) before the column binding step. They were fixed and imaged at 100× magnification or a 2D-STEDYCON setup at IGB of UIUC. EV RNA was isolated from eluted EVs (with or without RNaseA treatment) using the TriZOL reagent (Invitrogen) as per the user manual. Isolated RNA was subjected to cDNA synthesis using random or poly-A primers. PCR was carried out using mArc cDNA specific primers (**Table S2**) using Taq DNA master mix. PCR samples were thereby run on an agarose gel to confirm the presence of HaloArc mRNA inside EVs. Eluted EVs were lysed using RIPA buffer and incubated at 70˚C for 10 minutes. Lysed samples were mixed 1:50 with Bradford Plus Reagent (Thermo.). The estimated EV protein concentration was later used to treat primary neurons for GluA1 endocytosis assays (see below).

Negative-Stain Transmission Electron Microscopy of Arc capsids

Purified Arc protein was incubated with either cellular RNA extracted from HEK293T cells or RNA (of Arc or GFP) that was transcribed *in-vitro* using MAXIscript SP6 Kit (Invitrogen) at 7.3% w/w RNA-to-Arc ratio (10 nucleotides of RNA to 1 molecule of Arc). Once the RNA was added, the sample was diluted in high phosphate buffer (500 mM NaPO4, 0.5 mM EDTA, 50 mM Tris, pH 7.5) to 0.25 mg/mL and incubated at room temperature for 2 hours. A Copper 200-mesh grid coated with Formvar and carbon (Electron Microscopy Sciences Cat# FCF200CU50) was used

for negative staining. Grids were discharged for 25 seconds in a vacuum chamber at 15mA using the PELCO easiGlow 91000 Glow Discharge Cleaning System. A 5 µL sample was added to the grid. After 2 minutes, the excessive liquid was blotted using filter paper. The grid was dipped once in a 50-µL MiliQ water blob and then in a 50 µL 1% uranyl acetate blob on parafilm; the excessive solution was blotted gently in between with filter paper. Then, 5 µL of 1% uranyl acetate is added to the grid to stain for 30 seconds. The excessive stain was blotted and air-dried for 1 minute. All images were acquired under the JEOL JEM-1400 TEM in the Materials Research Laboratory Central Research Facilities, University of Illinois Urbana-Champaign.

Neuron surface GluA1 assay and analysis

DIV 14 mouse cortical neurons treated with EVs purified from wild-type or RalA/RalB double knockdown cells for four hours. After treatment, cells were washed once with PBS and fixed in 400 µL fixation buffer (4% paraformaldehyde and 4% sucrose in PBS) for 8 min in the 24-well plate. Cells were then washed twice with PBS (1 mL/ well) for 5 min each and blocked with 350 µL blocking buffer (1% BSA in PBS) for 1 hr at room temperature. The surface GluA1 was labeled by incubating with primary antibody against GluA1 (Millipore, MAB2263; 1:300 dilution) in 1% BSA at 4 °C overnight. Cells were washed again 3 times in PBS (1 mL/well, 10 min each) before incubated with the Alexa Fluor 488-conjugated secondary antibody (Thermo Scientific, A-11001; 1:200 dilution) for 2 hours at room temperature in the dark. The level of surface GluA1 is measured by fluorescence imaging on a confocal microscope.

Particle tracking and diffusion coefficient calculation

Particles were tracked from the HILO-acquired videos using the FIJI plugin TrackMate(76) (https://imagej.net/plugins/trackmate/). The *'spots.csv'* file generated in TrackMate was used for diffusion coefficient analysis. Briefly, all the tracks were exported from the spots file to a custom-written Python script (**Software S1**), which calculated the particle diffusion coefficient using the Co-variance Estimation (CVE) method. The diffusion coefficient (D_{CVE}) was calculated as follows:

$$D_{CVE,x} = \frac{(\Delta x_n)^2}{2\Delta t} + \frac{\Delta x_n + \Delta x_{n+1}}{\Delta t}$$

$$D_{CVE,y} = \frac{(\Delta y_n)^2}{2\Delta t} + \frac{\Delta y_n + \Delta y_{n+1}}{\Delta t}$$

where Δx_n is $_{xn+1}$-x_n in a trajectory time series where Δt is the integration time. The entire series of tracks was averaged out to get the final $D_{CVE,}$ as mentioned below.

$$D_{CVE} = \frac{Average(D_{CVE,x}) + Average(D_{CVE,y})}{2}$$

Particle fluorescence intensity was acquired as the maximum value of the intensity was acquired from each particle tracked by TrackMate. Colocalization for 2-color HILO videos was quantified by manual counting for all HaloArc clusters in the tracked videos. The quantification of colocalization in 2-color HILO videos involved manual counting of all HaloArc clusters present in the tracked videos to determine the frequency of overlap between the endosomal marker and HaloArc clusters. Briefly, each tracked HaloArc cluster was manually categorized as either overlapping (+) or not overlapping (-) with a HaloArc cluster. This process was repeated for all

three endosomal species tracked alongside HaloArc. The frequency of overlap was calculated using the following equation:

$$OverlapFrequency(HaloArc, marker) = \frac{No. of marker(+) HaloArc\ clusters}{Total\ HaloArc\ clusters\ observed}\ X\ 100$$

The fluorescence intensity of HaloArc carried in each kind of endosome was estimated as the maximum counts extracted from a HaloArc cluster throughout the tracked video. Step size histograms were generated as the distance between HaloArc clusters at every acquisition using the Trackmate-generated localizations from file '*spots.csv*' and calculated using the following equation:

$$Step - size(\Delta t) = \sqrt{[x(t + \Delta t) - x(t)]^2 + [y(t + \Delta t) - y(t)]^2}$$

Supplementary Figures

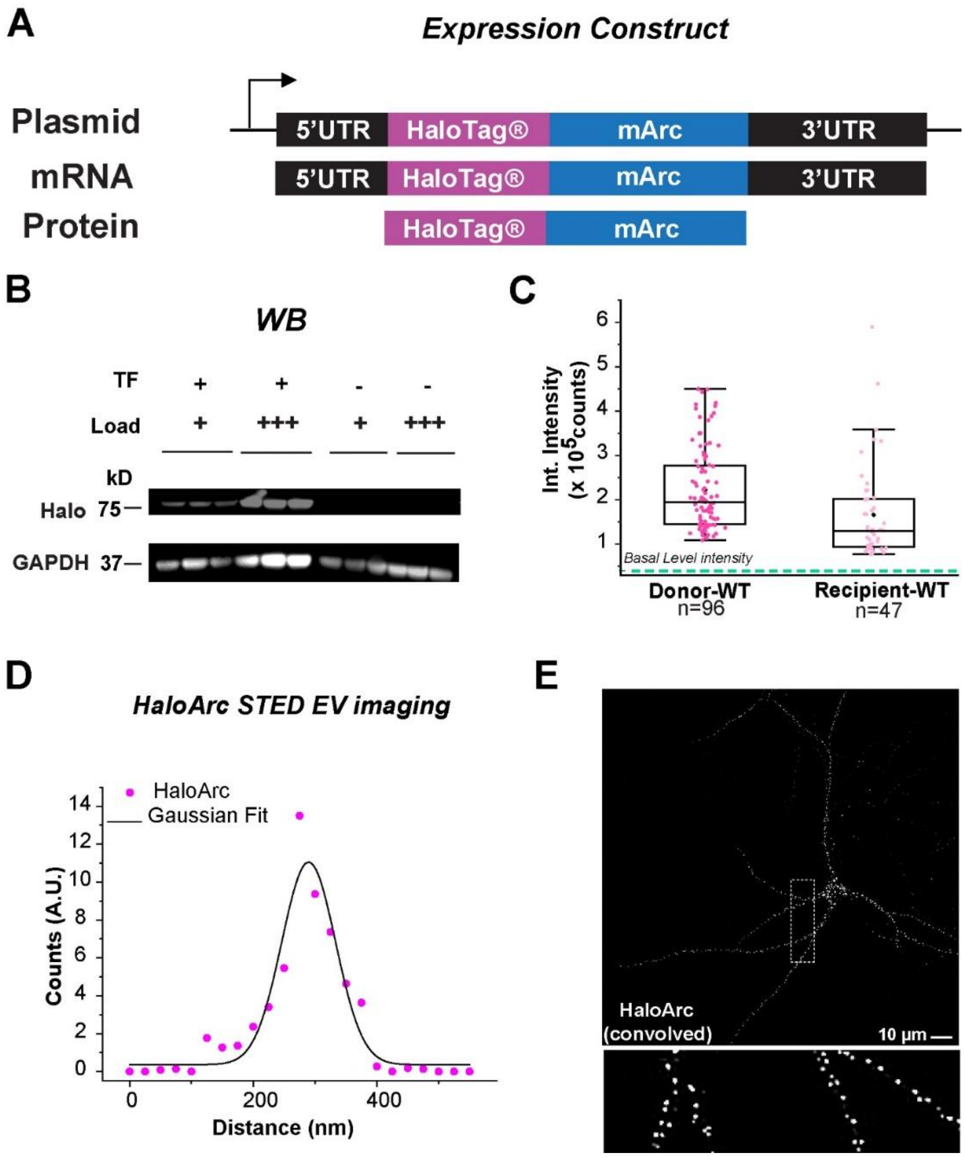

Fig. S1: A) Schematic representation of the plasmid construct used for expressing mArc in HEK-293T cells. The plasmid carries an N terminal HaloTag and untranslated regions on both the 5' and 3' ends of the RNA. B) Western blot analysis of cell lysates from HaloArc-transfected (TF+) or untransfected (TF-) cells. A 75 kDa band shows the expression of HaloArc in cells upon transfection. C) Quantification of fluorescence intensity of HaloArc in the donor and recipient cells. The green dashed line shows the basal intensity of untransfected cells. D) The intensity profile and one-dimensional Gaussian fit of a representative STED image of Arc capsid. E) A representative image of HaloArc-transduced primary rat cortical neurons showing the HaloArc cluster intensity after convolution. Warmer colors indicate higher intensities. The marked ROI is enlarged (bottom) to show neuronal processes carry bright clusters of HaloArc. Scale bar: 10 µm.

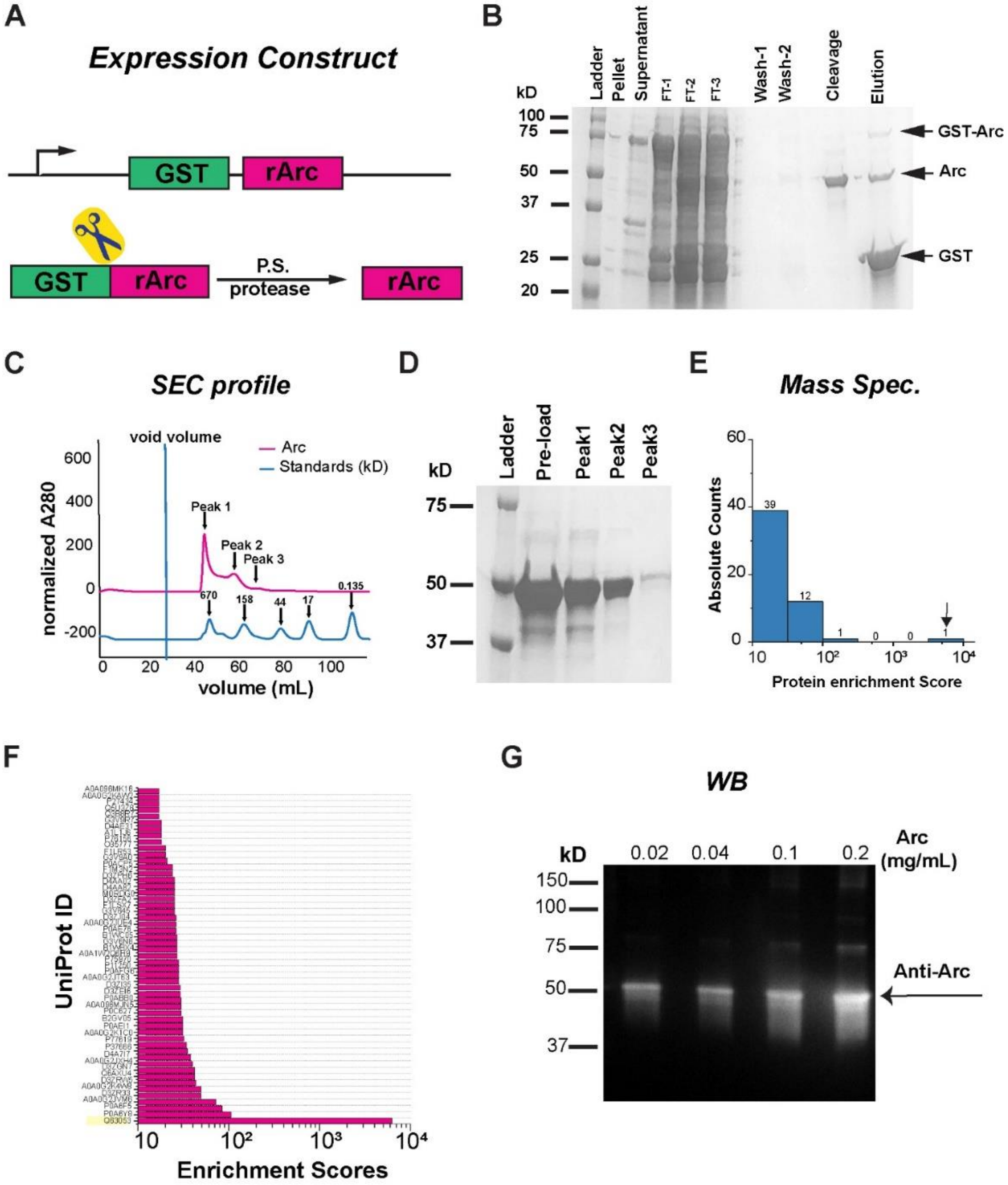

Fig. S2: Arc protein purification and characterization. **(A)** Schematic of the GST-Arc constructs used for affinity purification of Arc protein. GST tag is cleaved in the column with the PreScission (P.S.) protease. **(B)** GST-Arc overexpression in bacteria and affinity chromatography protein gel showing GST-Arc (75 kDa) was present mostly in the supernatant fraction after lysis. GST cleavage results in the elution of pure Arc protein (45 kDa). **(C)** Size exclusion chromatogram of purified Arc overlapped with protein standards showing Arc elutes in 3 different sizes of oligomers (peak 1, 2, and 3). **(D)** Protein gel for different SEC fractions collected showing a single 45 kDa band on SDS-PAGE. **(E)** Mass spectrometry results showing protein enrichment scores for the top 53 hits with $p<0.05$. **(F)** Individual scores identified Arc (uniport ID: Q63053) as the top hit. **(G)** Western blot analysis of purified Arc protein loaded with different doses.

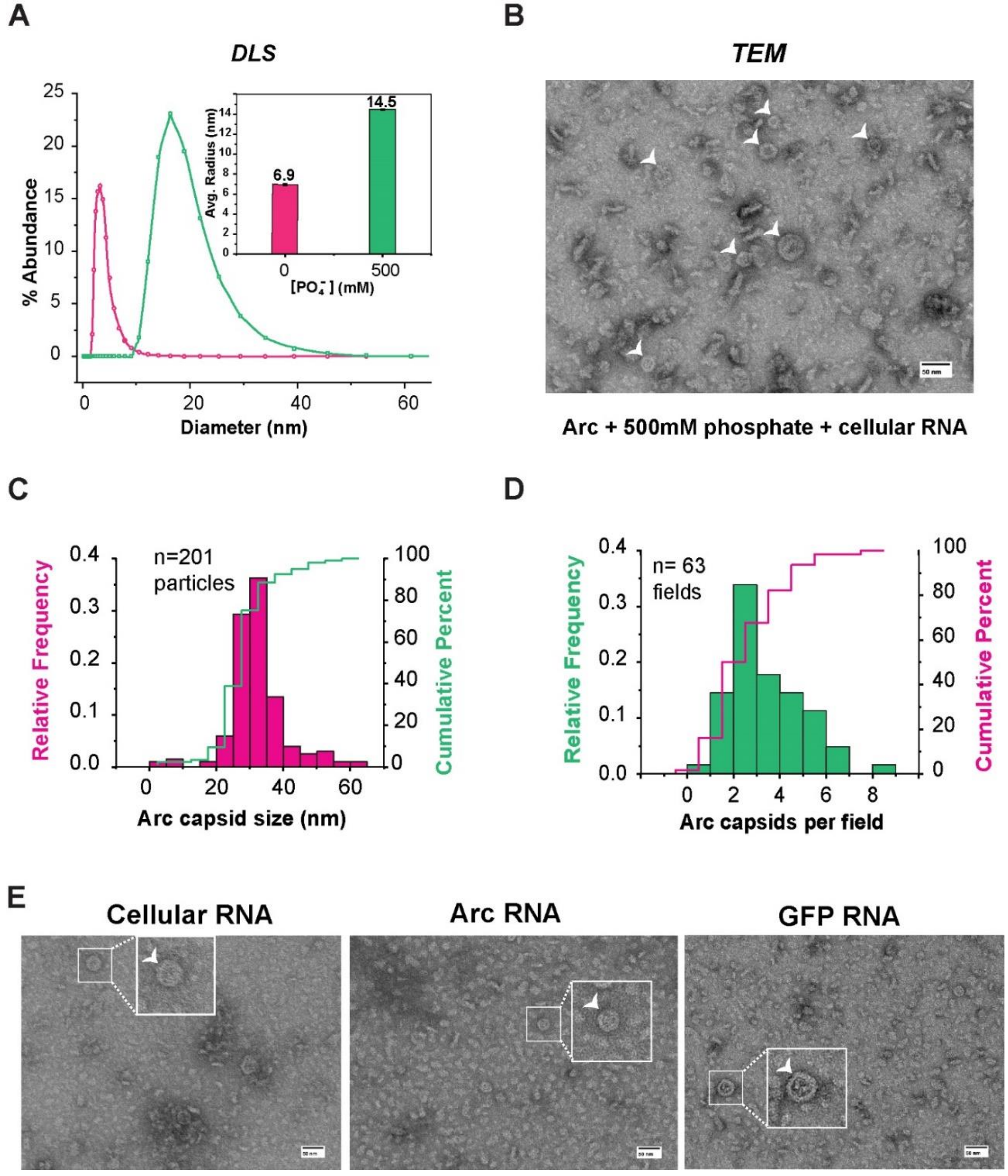

Figure S3. Purified Arc protein can assemble into capsids. (A) DLS analysis of Arc protein size with (green) and without (pink) high phosphate buffer treatment, 500 mM sodium phosphate. Inset: Average radius measured from DLS. **(B)** TEM micrograph of Arc capsids induced with RNA and phosphate buffer treatment of the purified Arc protein. Arrowheads mark the typical double-shelled capsid structure. Quantification of capsid diameter **(C)** (n=201 capsids; N=4 experiments) and the number of Arc capsids **(D)** per TEM field of view (n=63 field of view; N=4 experiments). **(E)** TEM micrographs of capsids induced under high phosphate conditions with cellular RNA (left), Arc RNA (middle) and GFP RNA (right). Insets show fully formed capsids captured under all conditions. Scale bar: 50 nm.

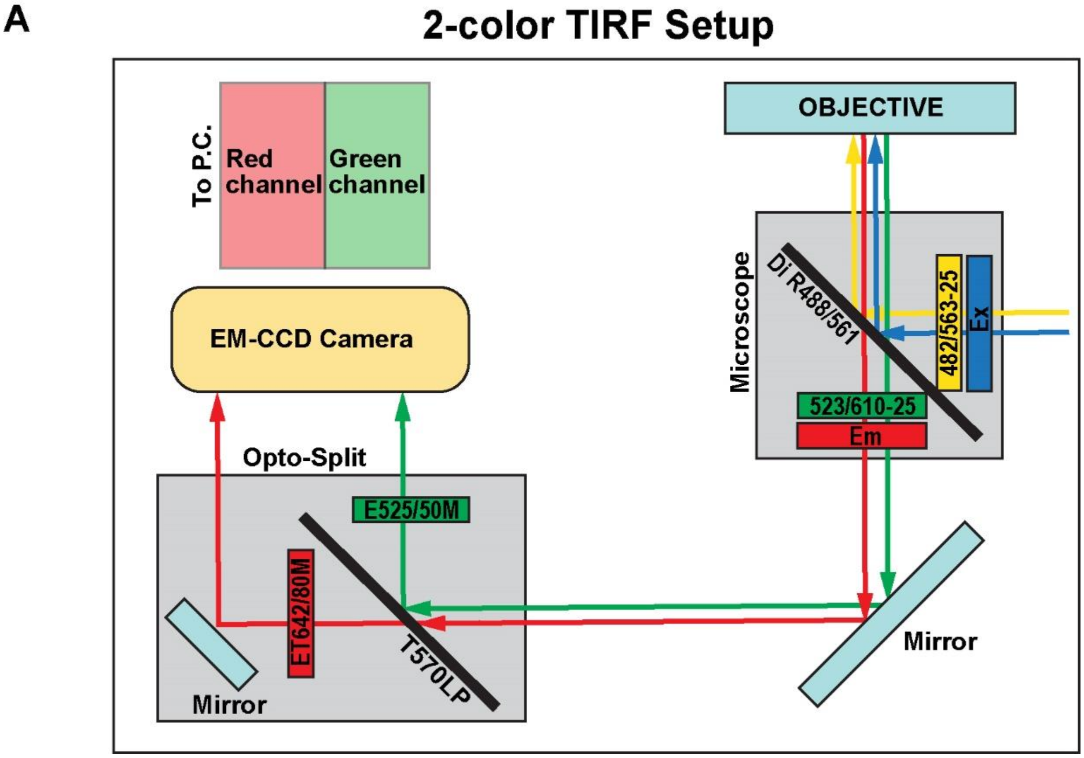

Figure S4: Scheme of two-color HILO microscopy. (A) Light path for the Opto-Split II setup for 2-color HILO microscopy. The incoming light is split into specific wavelengths and collected separately on the EM-CCD camera for simultaneous tracking of two fluorophores.

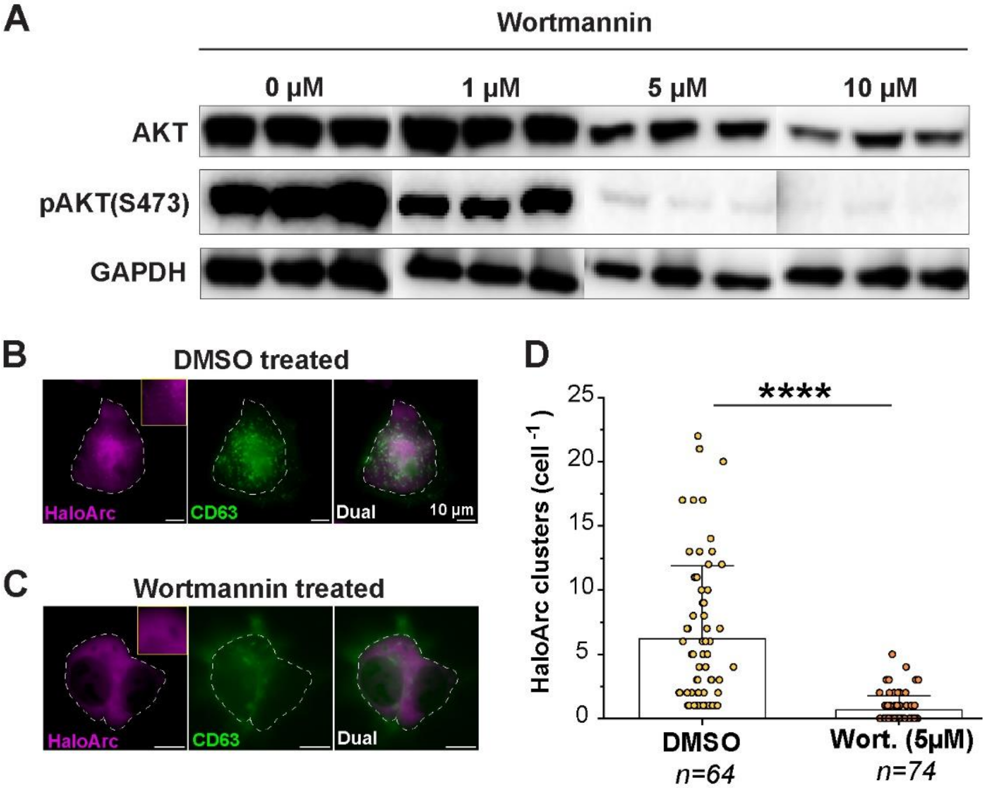

Figure S5: PI3K activity is necessary for Arc association to endosomal membranes. (A) Dose-response of Wortmannin-mediated PI3K inhibition probed by AKT phosphorylation at serine-473. **(B)** A representative epifluorescence image of a cell expressing HaloArc and CD63-sfGFP showing multiple HaloArc puncta. **(C)** Representative epifluorescence image of a cell expressing HaloArc and CD63-sfGFP after treatment with 5µM Wortmannin to inhibit PI3K activity showing loss of HaloArc puncta. **(D)** Quantification of HaloArc puncta in HEK293T cells with and without Wortmannin treatment (Mann-Whitney test, ***: p<0.001).

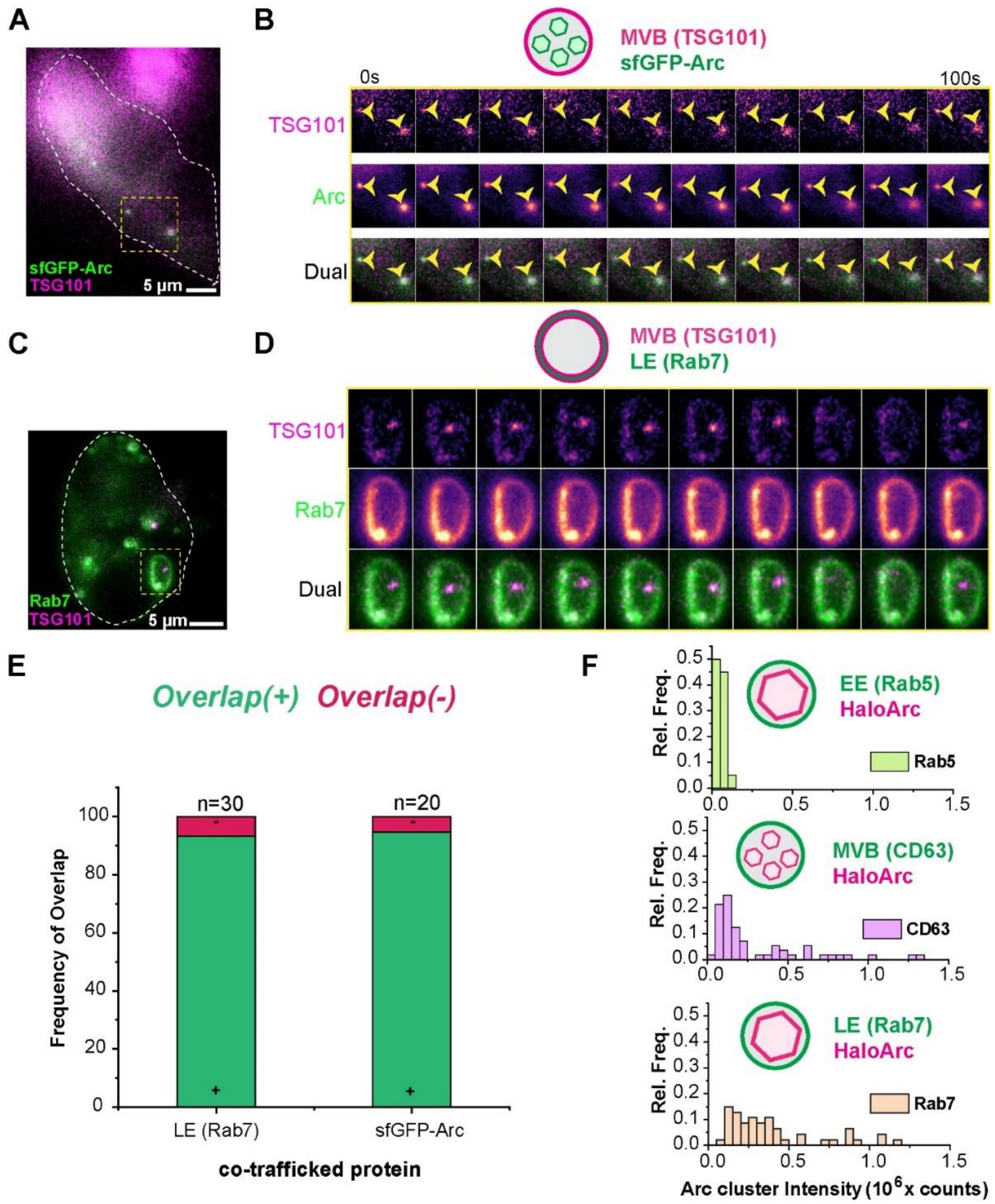

Figure S6: Arc colocalizes with endosomal and MVB markers in HEK293T cells. (A) A representative image of a HEK-293T cell co-expressing sfGFPArc (green) and mCherry-TSG101 (magenta) showing Arc clusters reside inside MVBs vesicles. **(B)** Time-lapse images of the selected ROI in A). Arrowheads mark Arc clusters. **(C)** A representative image of a HEK-293T cell co-expressing EGFP-Rab7 (green) and mCherry-TSG101 (magenta) showing MVB and late endosome markers overlap. **(D)** Time-lapse images of the selected ROI in C. **(E)** The frequency of overlap between mCherry-TSG101 and EGFP-Rab7 (left, n=30) or sfGFP-Arc (right, n=20). Green indicates overlapped, and red indicates non-overlapped puncta. **(F)** The fluorescence intensity distribution of HaloArc clusters residing inside early endosome (top, green), MVB (middle, pink) and late endosome (bottom, orange).

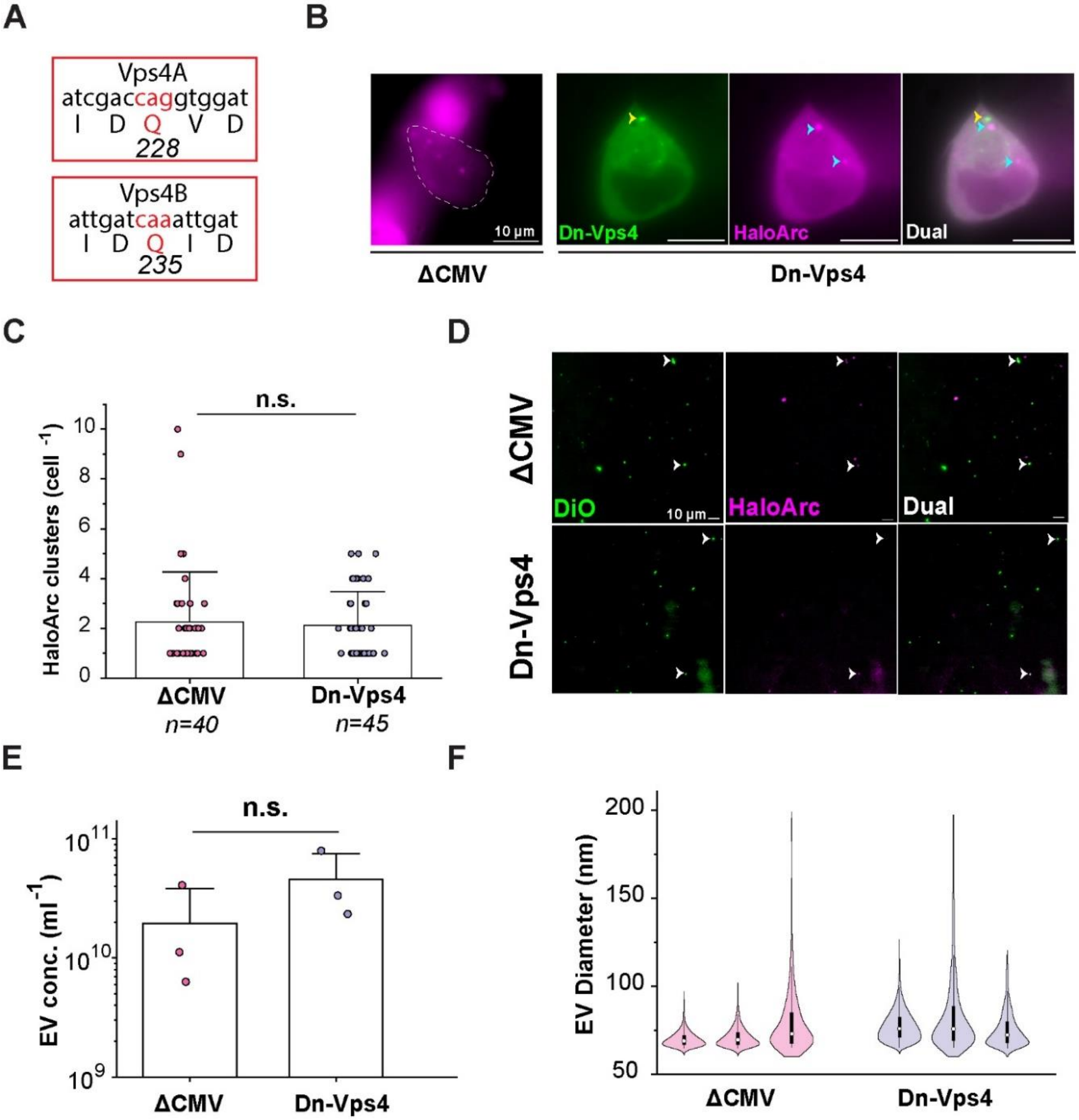

Figure S7: Dominant negative Vps4 does not impact HaloArc trafficking and secretion. HEK293T cells were co-transfected with HaloArc and negative control (ΔCMV) or dnVps4, followed by fluorescence labeling of HaloTag. **(A)** The sequence of dnVps4A (E228Q) and dnVps4B (E235Q) **(B)** Representative epifluorescence image of a HEK293T cell expressing HaloArc co-transfected with filler DNA (ΔCMV; left) and dominant-negative Vps4 plasmid (right) showing dn-Vps4 (yellow arrowheads) and HaloArc puncta (cyan arrowheads) do not overlap. **(C)** Quantification of HaloArc puncta in HEK293T cells shown in B. **(D)** Representative epifluorescence images of stained EVs purified from cells expressing HaloArc co-transfected with filler DNA (ΔCMV; top) or dominant-negative Vps4 plasmids (bottom). Scale bar in B and D: 10 μm. **(E)** Comparison of EV concentration from EVs in C. **(F)** Comparison of EV sizes from EVs in C. (Mann-Whitney test, n.s.: not significant).

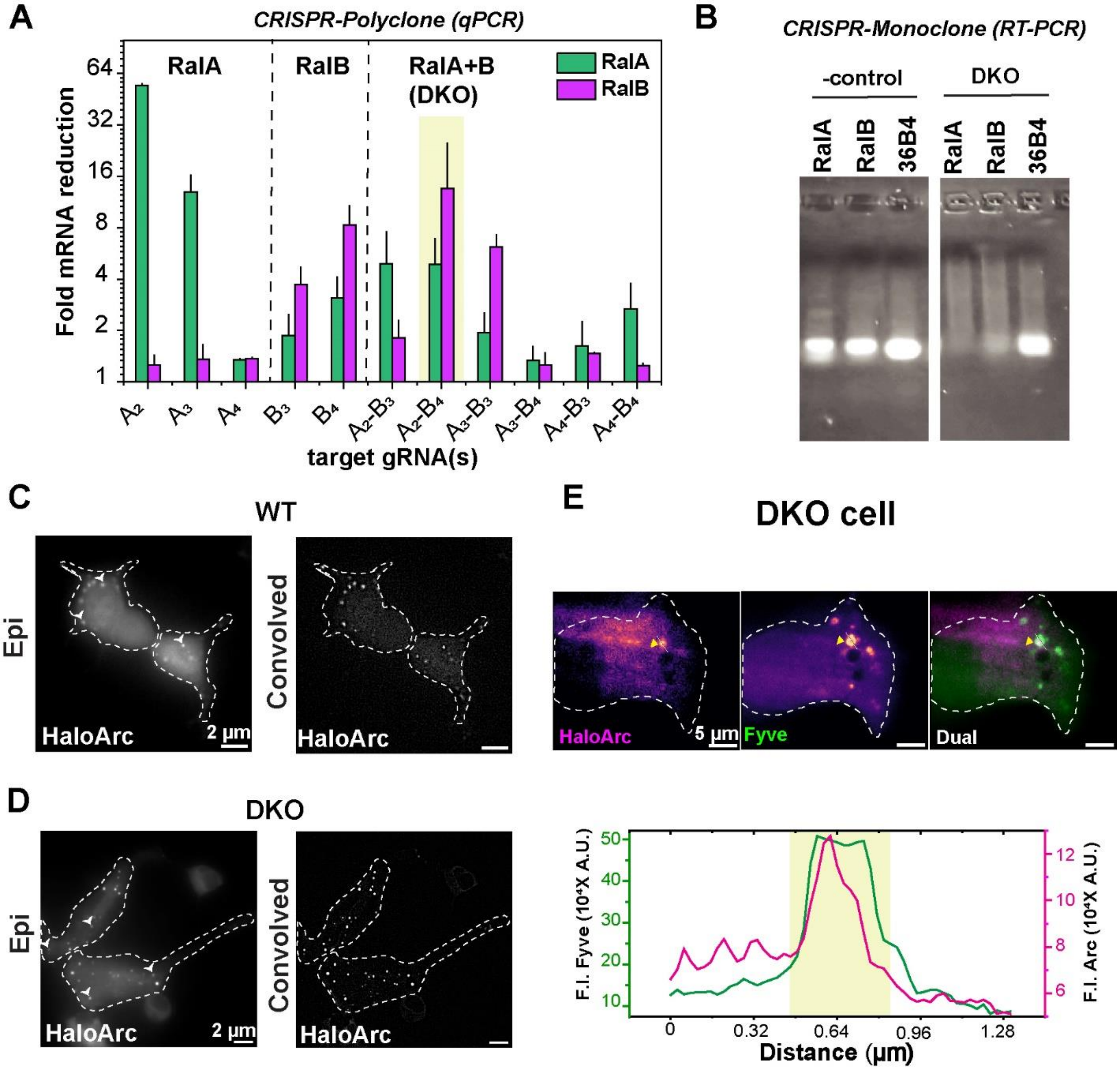

Figure S8: RalA/B double knockout leads to enlarged Arc puncta in HEK293T cells. (A) Relative fold reduction of RalA and RalB mRNA in polyclonal HEK293T cells treated with different gRNA combinations for CRISPR-Cas9 knockout using qPCR. Yellow inset highlights that gRNA pair 'A2-B4' showed a significant reduction in both RalA and RalB expression. **(B)** RT-PCR gel of selected monoclonal colony showing complete knockout of both RalA and RalB (right) compared to WT cells (left). 36B4 was used as an internal control for both A and B. Representative epifluorescence and convolved image of WT **(C)** and DKO **(D)** HEK293T cells expressing HaloArc Scale bar: 2 μm. **(E)** (top) A representative image of a DKO HEK-293T cell co-transfected with HaloArc and EGFP-2×FYVE. (bottom) The intensity profile across the line. The yellow highlight shows overlapping FYVE+ vesicle and Arc cluster. Scale bar: 5 μm.

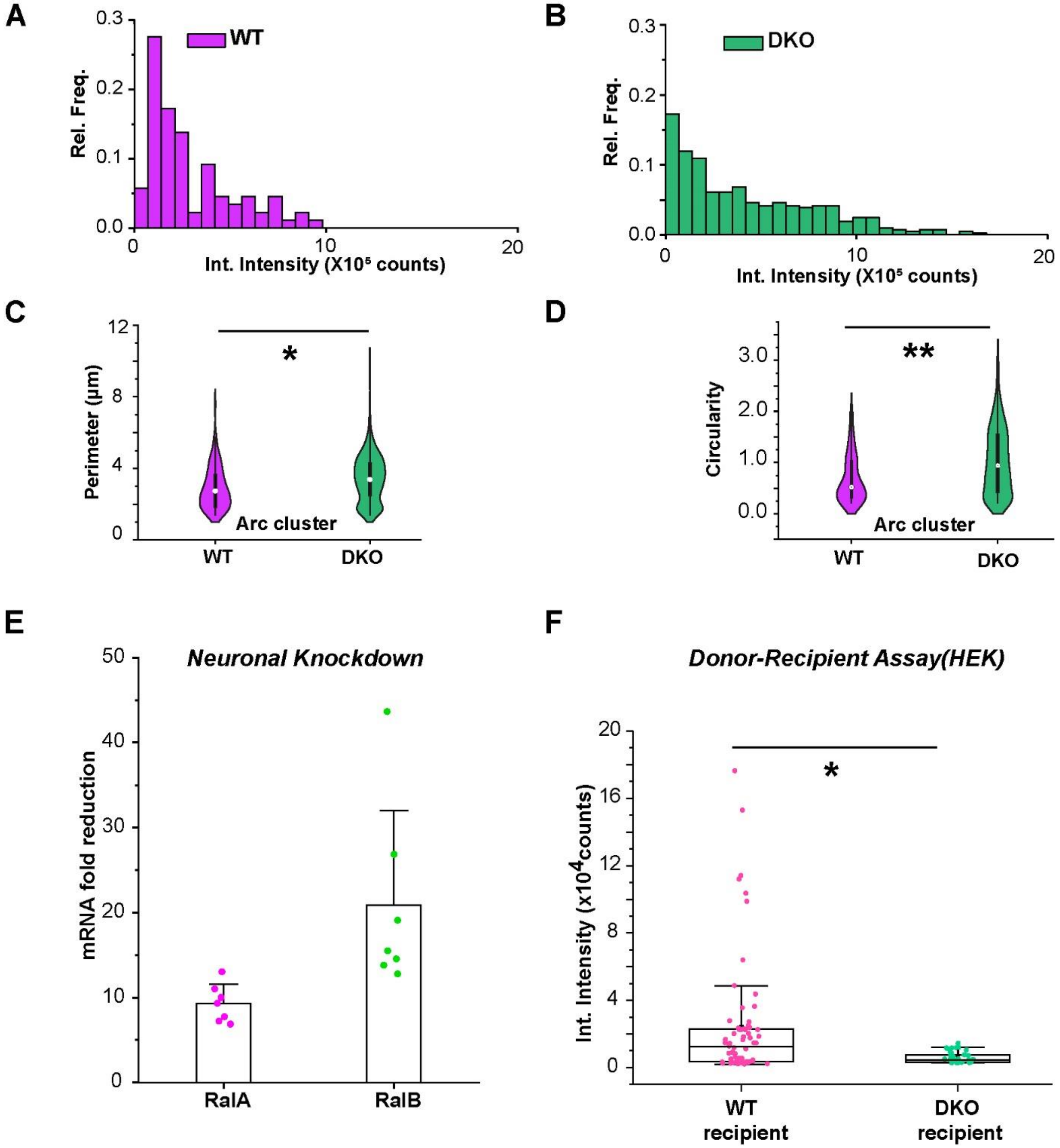

Fig. S9: RalA/B double knockout results in defective EV biogenesis and intercellular RNA transfer. Fluorescence intensity histogram of HaloArc expressed in wild-type **(A)** or RalA/B DKO **(B)** HEK293T cells. Comparison of perimeter **(C)** and circularity **(D)** of HaloArc clusters in both WT and DKO cells. **(E)** A qRT-PCR analysis confirming the knockdown of both RalA and RalB in rat cortical neurons. 36B4 gene served as the internal control gene. **(F)** HaloArc fluorescence intensities of recipient cells treated with the conditioned medium from HaloArc-expressing wild-type (left) and RalA/B DKO (right) cells. Mann-Whitney test; *: $p < 0.05$, **: $p < 0.01$.

Supplementary Tables

Table S1: Plasmids used in this work.

Name	Source
pCS2+-HaloTag-mArc-UTRs	This study
pCS2+-sfGFP-mArc-UTRs	This study
pCS2+-HaloTag	This study
pCS2+-sfGFP	This study
pEGFP-hRab5 (Kan)	Zhang Lab, UIUC
pEGFP-hRab7 (Kan)	Zhang Lab, UIUC
pMEH371 CD63 HA (amp)	Addgene #168220
pCS2+-sfGFP-hCD63 (Amp)	This study (Constructed from Addgene #168220)
pEF.6-mCherry-hTSG101 (Amp)	Addgene #38318
pEGFP-2×FYVE (Kan)	Addgene #140047
pEGFP-hMTMR1 (Kan)	This study (Constructed from Addgene #100723)
pEGFP-hINPP5E (Kan)	This study (Constructed from Addgene #171948)
pGEX-6p1-GST-rArcFL (Amp)	Prof. Jason Shepherd, Univ. of Utah
pLJM1-Halo-mArc-UTRs (Amp)	This study
pCS2+-SnapTag-mArc-UTRs (Amp)	This study (Constructed from Addgene #101135)
pEGFP-Vps4A(E228Q)	Addgene #80351
pEGFP-Vps4B(E235Q)	This study (Constructed from Addgene #169320)

Table S2: Primers used in this work.

S. No.	Name	Sequence	Purpose
1	h36B4-RT Fw	GTGTTCGACAATGGCAGCAT	KO in HEK-293T (qPCR)
2	h36B4-RT Rv	GACACCCTCCAGGAAGCGA	KO in HEK-293T(qPCR)
3	hRalA-RT Fw	ATGGCTGCAAATAAGCCCAAG	KO in HEK-293T(qPCR)
4	hRalA-RT Rv	TGTCTGCTTTGGTAGGCTCATA	KO in HEK-293T(qPCR)
5	hRalB-RT Fw	AGCCCTGACGCTTCAGTTC	KO in HEK-293T(qPCR)
6	hRalB-RT Rv	AGCGGTGTCCAGAATATCTATCT	KO in HEK-293T(qPCR)
7	mArc-RT Fw	GGTAAGTGCCGAGCTGAGATG	EV expression (RT-PCR)
8	mArc-RT Rv	CGACCTGTGCAACCCTTTC	EV expression(RT-PCR)
9	m36B4-RT Fw	TGAGATTCGGGATATGCTGTTGG	EV expression(RT-PCR)
10	m36B4-RT Rv	CGGGTCCTAGACCAGTGTTCT	EV expression(RT-PCR)
11	rRalA-RT Fw	ATGGCTGCAAACAAGCCCAAG	KD in cortical neurons
12	rRalA-RT Rv	TAGCTGTCTGCTTTGGTAGGTTC	KD in cortical neurons (qPCR)
13	rRalB-RT Fw	GAGAAGGCTTTCTGCTGGTGTTC	KD in cortical neurons(qPCR)
14	rRalB-RT Rv	ACGACCAGCAGAGGGATTTTGTC	KD in cortical neurons(qPCR)
15	r36B4-RT Fw	CGAGATTAGGGACATGCTGCTGG	KD in cortical neurons(qPCR)
16	r36B4-RT Rv	AGACCGGTGTTCTGAGCAGGTAC	KD in cortical neurons(qPCR)
17	gRalA_1 Fw	CACCGCAACTACTTCCGAAGTGGGG	KO in HEK-293T
18	gRalA_1 Rv	AAACCCCCACTTCGGAAGTAGTTGC	KO in HEK-293T
19	gRalA_2 Fw	CACCGTCTACAGTTCATGTACGATG	KO in HEK-293T
20	gRalA_2 Rv	AAACCATCGTACATGAACTGTAGAC	KO in HEK-293T
21	gRalA_3 Fw	CACCGAGACAACTACTTCCGAAGTG	KO in HEK-293T
22	gRalA_3 Rv	AAACCACTTCGGAAGTAGTTGTCTC	KO in HEK-293T
23	gRalA_4 Fw	CACCGGCAGTGGAATGTTAACTACG	KO in HEK-293T
24	gRalA_4 Rv	AAACCGTAGTTAACATTCCACTGCC	KO in HEK-293T
25	gRalB_1 Fw	CACCGCGGGCCAACGTGGACAAGGT	KO in HEK-293T
26	gRalB_1 Rv	AAACACCTTGTCCACGTTGGCCCGC	KO in HEK-293T

27	gRalB_2 Fw	CACCGTTGTTTCCCACGACGAGCAG	KO in HEK-293T
28	gRalB_2 Rv	AAACCTGCTCGTCGTGGGAAACAAC	KO in HEK-293T
29	gRalB_3 Fw	CACCGCGAGATAACTACTTTCGGAG	KO in HEK-293T
30	gRalB_3 Rv	AAACCTCCGAAAGTAGTTATCTCGC	KO in HEK-293T
31	gRalB_4 Fw	CACCGCATACATGAACTGAAGCGTC	KO in HEK-293T
32	gRalB_4 Rv	AAACGACGCTTCAGTTCATGTATGC	KO in HEK-293T
33	r-mRalA1 Fw	CCGGctgacctgaccatactaataaCTCGAGtt attagtatggtcaggtcagTTTTTG	KD in cortical neurons
34	r-mRalA1 Rv	AATTCAAAAActgacctgaccatactaataaCT CGAGttattagtatggtcaggtcag	KD in cortical neurons
35	r-mRalA2 Fw	CCGGgacctgaccatactaataaatCTCGAGat ttattagtatggtcaggtcTTTTTG	KD in cortical neurons
36	r-mRalA2 Rv	AATTCAAAAAgacctgaccatactaataaatCT CGAGatttattagtatggtcaggtc	KD in cortical neurons
37	r-mRalB1 Fw	CCGGaaGCCGACAGTTACAGAAAGAC TCGAGTCTTTCTGTAACTGTCGGCttT TTTTG	KD in cortical neurons
38	r-mRalB1 Rv	AATTCAAAAAaaGCCGACAGTTACAG AAAGACTCGAGTCTTTCTGTAACTGT CGGCtt	KD in cortical neurons
39	r-mRalB2 Fw	CCGGctgttcccttcatacttaaatCTCGAGattta agtatgaagggaacagTTTTTG	KD in cortical neurons
40	r-mRalB2 Rv	AATTCAAAAActgttcccttcatacttaaatCTC GAGatttaagtatgaagggaacag	KD in cortical neurons

Table S3: Reagents used in this work.

S. No.	Name	Brand	Identifier
vectors			
1	pCS2+ vector	NovoPro	V011726
2	pLenti-CRISPRv2 vector	Addgene	52961
3	pLKO.1 TRC cloning vector	Addgene	10878
4	pLJM1 vector	Addgene	91980
enzymes/Master-mixes			
5	FD-EcoRI	Thermo Fisher Scientific	FD0274
6	FD-NotI	Thermo Fisher Scientific	FD0593
7	FD-PacI	Thermo Fisher Scientific	FD2204
8	FD-BcuI	Thermo Fisher Scientific	FD1254
9	FD-BshTI	Thermo Fisher Scientific	FD1464
10	T4-polynucleotide kinase (T4-PNK)	NEB	M0201
11	FD-EspIII	Thermo Fisher Scientific	FD0454
12	Shrimp Alkaline phosphatase (rSAP)	NEB	M0371
13	Infusion cloning master mix	Takara Bio	638944
14	Reverse Transcriptase	NEB	M0253
15	SYBR green master mix	Applied Biosystems	A25742
16	Go-Taq green master mix	Promega	M7122
17	RNase	Thermo Fisher Scientific	EN0531
18	PreScisson Protease	Cytiva	27-0843-01
Cell culture reagents			
19	Dulbecco's Modified Eagle Media	Corning	10-014-CM
20	Penicillin-streptomycin solution 100X	Corning	30-002-CI
21	Fetal bovine serum	Sigma Aldrich	F2442

22	PEImax	PolySciences	24765
23	Dulbecco's Modified Phosphate Buffered Saline	Corning	20-030-CV
24	Phenol red-free DMEM	Gibco	31053028
25	Trypsin	Life Tech	15050065
Antibodies			
26	Anti-HaloTag	Promega	G9211
27	Anti-Arc	Synaptic Systems	156 003
28	Anti-GAPDH	Cell Signaling Technologies	2118L
29	Anti-MAP2	Abcam	Ab5392
30	Chicken secondary (Alexa-647)	Abcam	Ab150175
31	Mouse secondary HRP	Cell Signaling Technologies	7076P2
32	Rabbit secondary HRP	Cell Signaling Technologies	7074S
Buffers/Reagents			
33	RIPA buffer	Millipore Sigma	R0278
34	4X NU-PAGE	Invitrogen	NP0007
35	BME	MP Biomedicals	0219483425
36	Bradford Reagent	Thermo Fisher Scientific	23200
37	SDS buffer	Biorad	1610732
38	SDS-PAGE gel	Biorad	4561043
39	Clarity ECL WB substrate	Biorad	170-5060
40	Agarose	Fisher	BP160100
41	PVDF membrane	Biorad	1620177
42	Copper 200-mesh grids	Electron Microscopy Sciences	FCF200CU50
43	TriZOL reagent	Invitrogen	15596018
44	Chloroform	Thermo Fisher Scientific	C298
45	Glycogen	Invitrogen	10814010

46	DEPC water	Invitrogen	462224
47	Ethanol	Decon Labs	VN1170
48	Isopropanol	VWR	3032-16
49	DTT	Thermo Scientific	20290
50	PMSF	Thermo Scientific	36978
51	LB broth	Fisher Scientific	BP9723
52	L-Glutathione	Fisher Scientific	AAJ6216606
53	PIP-strip	Echelon Biosciences	P-6001
54	Wortmannin	Millipore Sigma	681675
Kits			
55	GeneJet RNA purification kit	Thermo Fisher Scientific	K0731
56	Capturem EV isolation mini kit	Takara Bio	635741
57	ExoEasy Maxi Kit	Qiagen	76064
58	GeneJet plasmid miniprep	Thermo Fisher Scientific	K0502
59	GeneJet Gel extraction kit	Thermo Fisher Scientific	K0691
60	MaxiScript SP6 Kit	Invitrogen	AM1340
Dyes/Fluorophores			
61	JF-549 halo ligand	Promega	GA1110
62	JF-646 halo ligand	Promega	GA1120
63	Vybrant DiO cell-labeling solution	Thermo Fisher Scientific	V22886
64	AlexaFluor-647 Snap substrate	NEB	S9136S
Antibiotics			
65	Puromycin	Gold Biotechnology	P-600-100
66	Ampicillin	Fisher Scientific	bp176025
67	Kanamycin	Fisher Scientific	bp9065

Supplementary Movie Legends

Movie S1 (separate file). **Representative time-stamped HILO imaging of a HEK293T cell expressing HaloArc showing intracellular HaloArc clusters into slow diffusing puncta.** Cells are labeled with 200nM JF549 HaloTag ligand. Warmer colors indicate higher intensities. Related to Fig. 2A.

Movie S2 (separate file). **Representative time-stamped HILO imaging of a HEK293T cell expressing HaloTag showing fast diffusing intracellular Halotag molecules.** Cells are labeled with 200nM JF549 HaloTag ligand. Warmer colors indicate higher intensities. Related to Fig. 2B.

Movie S3 (separate file). **Representative time-stamped HILO imaging of a WT HEK293T cell co-expressing HaloArc (magenta) and EGFP-2×FYVE (green) showing colocalization of Arc clusters with PI3P carrying vesicles.** Cells are labeled with 200nM JF549 HaloTag ligand. Related to Fig. 3C.

Movie S4 (separate file). **Representative time-stamped HILO imaging of a DKO HEK293T cell co-expressing HaloArc (magenta) and EGFP-2×FYVE (green) showing colocalization of Arc clusters with PI3P carrying vesicles.** Cells are labeled with 200 nM JF549 HaloTag ligand. Related to Fig. S8E.

Movie S5 (separate file). **Representative time-stamped HILO imaging of a HEK293T cell co-expressing HaloArc (magenta) and EGFP-Rab5 (green) showing localization of Arc clusters with early endosomes.** Cells are labeled with 200nM JF549 HaloTag ligand. Related to Fig. 4A.

Movie S6 (separate file). **Representative time-stamped HILO imaging of a HEK293T cell co-expressing HaloArc (magenta) and sfGFP-CD63 (green) showing localization of Arc clusters with MVBs.** Cells are labeled with 200nM JF549 HaloTag ligand. Related to Fig. 4B.

Movie S7 (separate file). **Representative time-stamped HILO imaging of a HEK293T cell co-expressing HaloArc (magenta) and EGFP-Rab7 (green) showing localization of Arc clusters with late endosomes and MVBs.** Cells are labeled with 200nM JF549 HaloTag ligand. Related to Fig. 4C.

Movie S8 (separate file). **Representative time-stamped HILO imaging of a HEK293T cell co-expressing sfGFP-Arc (green) and mCherry-TSG101 (magenta) showing Arc clusters also**

localize with ESCRT protein TSG101. Cells are labeled with 200nM JF549 HaloTag ligand. Related to Fig. S6A-B.

Movie S9 (separate file). **Representative time-stamped HILO imaging of a HEK293T cell co-expressing MVB marker mCherry-TSG101 (magenta) and EGFP-Rab7 (green) showing late endosomes and MVBs colocalize.** Cells are labeled with 200nM JF549 HaloTag ligand. Related to Fig. S6C-D.

Supplementary Dataset

Dataset S1 (separate file). Diffusion Coefficient values for HaloArc and HaloTag tracked particles using the Co-variance estimator method.

Dataset S2 (separate file). Step size and fluorescence intensities of Arc clusters colocalizing with EE, MVB and LE.

Dataset S3 (separate file). WT and DKO Arc cluster analysis data for fluorescence intensity, cluster number per cell, integrated intensity, perimeter and circularity.

Software S1 (separate file). A custom-written script for diffusion coefficient analysis was performed in the paper using the CVE method. https://github.com/kritikamehta1794/Diffusion-coefficient_CVE.git
